# Spine nanostructure profiling of cultured neurons from mouse models reveals a schizophrenia-linked role for Ecrg4

**DOI:** 10.7554/eLife.109083

**Published:** 2026-08-11

**Authors:** Yutaro Kashiwagi, Qingrui Liu, Yasuhiro Go, Ryo Saito, Atsu Aiba, Takanobu Nakazawa, Shigeo Okabe

**Affiliations:** 1 Department of Cellular Neurobiology, Graduate School of Medicine, The University of Tokyo Tokyo Japan; 2 Graduate School of Information Science, University of Hyogo Kobe Japan; 3 National Institute for Physiological Sciences (NIPS), National Institutes of Natural Sciences Okazaki Japan; 4 Exploratory Research Center on Life and Living Systems (ExCELLS), National Institutes of Natural Sciences Okazaki Japan; 5 Laboratory of Animal Resources, Center for Disease Biology and Integrated Medicine, Graduate School of Medicine, The University of Tokyo Tokyo Japan; 6 Department of Bioscience, Faculty of Life Sciences, Tokyo University of Agriculture Tokyo Japan; 7 Laboratory for Imaging Neural Dynamics, RIKEN Center for Brain Science Saitama Japan; University of Oxford United Kingdom; Stanford Medicine United States

**Keywords:** synapse, dendritic spine, super-resolution microscopy, autism spectum disorder, schizophrenia, Ecrg4, Mouse

## Abstract

Dendritic spine dysfunction may contribute to the etiology and symptom expression of neuropsychiatric disorders. The intimate relationship between spine morphology and function suggests that decoding disease-related abnormalities from spine morphology can aid in developing synapse-targeted interventions. Here, we describe a population analysis of dendritic spine nanostructure applied to the objective grouping of multiple mouse models of neuropsychiatric disorders. This method has identified two major groups of spine phenotypes linked to schizophrenia and autism spectrum disorder (ASD). An increase in spine subpopulation with small volumes characterized the spines of schizophrenia-associated mouse models, whereas a spine subset with large volumes increased in ASD models. Schizophrenia-associated mouse models showed higher similarity in spine morphology, driven by reduced size and growth of nascent spines. The expression of *Ecrg4*, a gene encoding small secretory peptides, was increased in schizophrenia-associated mouse models, and functional studies confirmed its critical involvement in impaired spine dynamics and shape. These results suggest that population-level spine analysis provides rich insights into heterogeneous spine pathology, facilitating the identification of new molecular targets related to core synaptic dysfunction.

## Introduction

Information processing in the neocortex and hippocampus relies on fast excitatory synaptic transmission between pyramidal neurons. Many excitatory synapses that release glutamate project onto tiny structures protruding from dendritic shafts termed spines, and the morphological properties of these spines strongly influence synaptic transmission and function, including the activity-dependent synaptic plasticity implicated in learning and memory (Rochefort and Konnerth, 2012; Sala and Segal, 2014). In general, larger spines contain a greater number of glutamate receptors and are functionally stronger (Matsuzaki et al., 2001). The morphological parameters defining spine shape, such as length, radius, and volume, are also highly dynamic. For instance, small nascent spines can be enlarged by high-frequency stimuli, inducing long-term potentiation (LTP) of synaptic transmission (Matsuzaki et al., 2004; Nägerl et al., 2004); alternatively, spine volume can be reduced by low-frequency stimuli that induce long-term depression (LTD) (Nägerl et al., 2004; Zhou et al., 2004). Due to these dynamic changes, spines on pyramidal neurons exhibit substantial morphological variation, even within the same dendritic branch. In addition to direct synaptic activation inducing LTP or LTD, spine morphology can be altered by cyclical hormonal changes, neuromodulators, and various activity-independent processes (Loewenstein et al., 2011; Loewenstein et al., 2015; Statman et al., 2014; Hazan and Ziv, 2020). In turn, these changes in spine morphology are associated with marked changes in neural function and behavior.

Recent advances in super-resolution microscopy have provided new opportunities for the study of spine structural diversity and dynamics. An investigation using stimulated emission depletion (STED) imaging reported an increase in spine neck size after LTP, resulting in stronger electrical coupling between the spine head and the dendritic shaft (Tønnesen et al., 2014). A structured illumination microscopy (SIM) imaging study of cultured hippocampal neurons revealed expansion of the spine head convexity after LTP induction (Kashiwagi et al., 2019), and suggested that this structural transformation may increase the adhesion of presynaptic and postsynaptic membranes. SIM imaging has also been applied to record the dynamic properties of spinules (Zaccard et al., 2020), thin protrusions extending from existing spines that may function in secondary synapse formation. Collectively, these reports implicate spine nanostructural dynamics in multiple core synaptic properties and functions, including neurotransmission, mechanical stability, synaptogenesis, and plasticity.

Genetic mutations and variants that disrupt synaptic function are strongly implicated in the pathogenesis of neuropsychiatric disorders such as autism spectrum disorder (ASD) and schizophrenia (Penzes et al., 2011; Fromer et al., 2014; Rodriguez-Murillo et al., 2012; Gulsuner et al., 2013; Bourgeron, 2015; Terashima et al., 2022; Zieger and Choquet, 2021). Based on these genomic aberrations, numerous mouse models of ASD and schizophrenia have been established and subsequently shown to harbor abnormalities in both spine morphology and physiology (Terashima et al., 2022; Yilmaz et al., 2021; Obi-Nagata et al., 2023; Mukai et al., 2008; Nakai et al., 2018). Moreover, several studies have reported synaptic dysfunction in neurons differentiated from patient-derived pluripotent stem cells (Shcheglovitov et al., 2013; Llamosas et al., 2020; Yamamoto et al., 2021; Wen et al., 2014). Schizophrenia and ASD share several clinical features, suggesting common genetic risk factors and etiology (Chisholm et al., 2015; De Crescenzo et al., 2019). However, a diametrical model emphasizes the distinct social-cognitive properties of these two major psychiatric disorders (Nenadić et al., 2021; Crespi and Badcock, 2008). Both ASD and schizophrenia also show highly heterogeneous clinical phenotypes, potentially stemming from distinct pathogenic processes within disease categories. Therefore, in addition to clinical features, comparative biological phenotyping of multiple ASD and schizophrenia models may reveal shared as well as disease-specific pathogenic processes.

Genetic studies on ASD and schizophrenia have identified copy-number variants and protein-disrupting single-nucleotide variants that confer higher disease risk, and mouse models harboring these genetic abnormalities frequently exhibit behavioral and neurological features resembling those of the clinical conditions (termed endophenotypes) (Terashima et al., 2022; Pratt et al., 2012). However, only a limited number of studies objectively compare phenotypes related to synaptic structure and function across disease models. An in vivo two-photon imaging study reported accelerated spine turnover in three genetically distinct mouse models of ASD (Isshiki et al., 2014). Also, multiple mouse models of ASD were found to exhibit a similar imbalance in the activity of excitatory and inhibitory neurons (Lee et al., 2017). These studies support the idea that distinct genetic mutations may induce similar synaptic and circuit phenotypes in ASD-associated mouse models. However, differences in synaptic properties between ASD and schizophrenia have not yet been comprehensively examined.

In this study, we developed an objective method for identifying population-level differences in spine nanostructure. This method, which has been applied to multiple mouse models of psychiatric disorders, identified two distinct groups corresponding to ASD and schizophrenia. An increase in a specific spine subpopulation with small volumes characterized the spines of mouse models of schizophrenia. In turn, ASD-related mouse models showed an opposite spine phenotype. The schizophrenia-related phenotype was associated with reduced size and growth of nascent spines and enhanced spine turnover. Further gene expression analysis identified the overexpression of *Ecrg4*, a gene encoding a precursor of hormone-like peptides, as a candidate mediator of this schizophrenia-associated spine phenotype. Population-level analysis of spine nanostructures is a powerful approach for understanding heterogeneous synaptic impairments in psychiatric disorders.

## Results

### Spine nanostructure imaging and generation of the spine density plot

**Figure 1.**
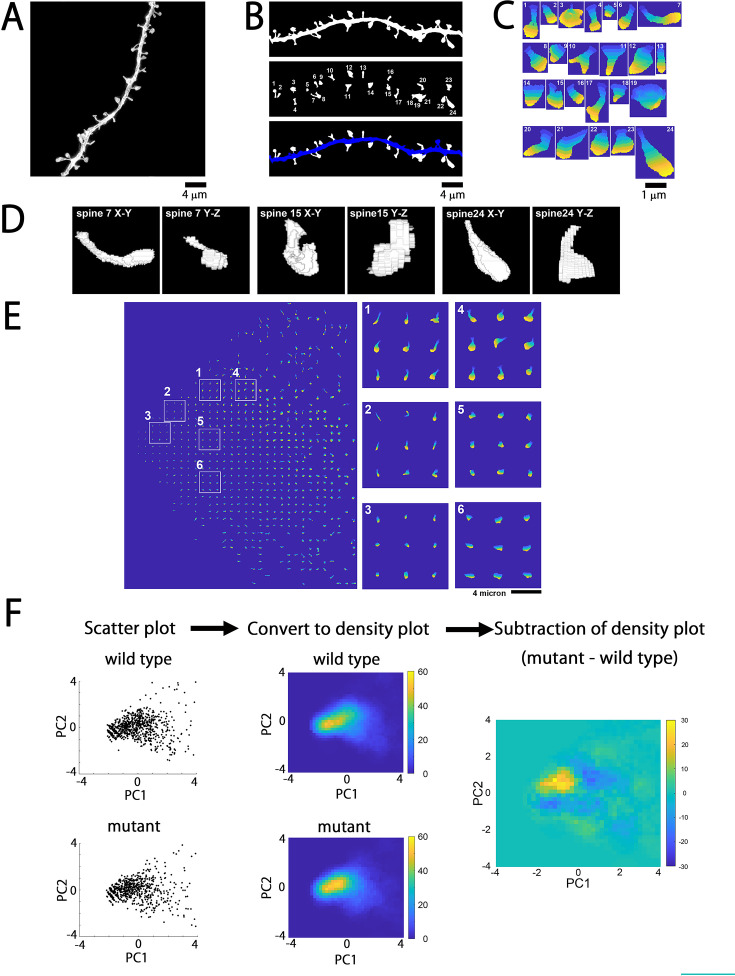
Structured illumination microscopy (SIM) imaging of dendritic spines, automated measurements of spine morphology, and generation of subtracted spine density plots for population-level analysis. (**A**) Original SIM image of a dendritic shaft stained with lipophilic membrane dye DiI. (**B**) Binarized image of the same dendrite (top) and segmented spines numbered from 1 to 24 (middle). The bottom image shows segmented spines (white) and the dendritic shaft (blue). (**C**) Enlarged images of the individual spines shown in (**B**). The pseudocolor images indicating the relative positions of the spine segments from the base (blue) to the tip (yellow). (**D**) 3D views of three spines (Nos. 7, 15, and 24) viewed from different angles. (**E**) Principal component analysis (PCA)-based dimensional reduction of spine characteristics plotted in the plane of principal components (PC)1 and PC2. (**F**) Process of generating a subtracted density plot. The scatter plot of spine distribution in the PC1–PC2 plane based on morphological parameters was converted into a density plot for each culture source (genotype or treatment group), and the corresponding plots were subtracted to reveal differences in spine morphology at the population level. Bars, 4 μm for A, B, and E, 1 μm for C.

We have designed a method for objectively comparing spine properties among multiple mouse models of psychiatric disorders at the population level using SIM imaging. Previous results demonstrated that high-resolution SIM images of dendritic spines in cultured hippocampal neurons can provide reliable information about spine size and morphological properties (Kashiwagi et al., 2019). In the current study, the analytical procedures for SIM images were further improved for quantitative comparisons. Three-dimensional SIM images of dendritic spines were captured in DiI-stained cultured neurons derived from control and disease model mouse hippocampus (Figure 1A). This staining procedure provided a better image quality than fluorescent protein-based labeling because of the higher fluorescence signal from the plasma membrane. SIM images were processed using custom-made scripts to identify and segment the individual dendritic spines (Figure 1B–D). We previously reported a set of scripts designed to isolate dendritic spines automatically (Kashiwagi et al., 2019). However, the segmentation accuracy still necessitated manual inspection. In the newly developed scripts, we introduced a step to readjust the spine–shaft boundary, enabling fully automated measurement of multiple morphological parameters, including length, surface area, and volume. In addition, the isolated spines were further divided into 160-nm-thick longitudinal segments, yielding a total of 64 shape descriptors.

**Figure 1—figure supplement 1.**
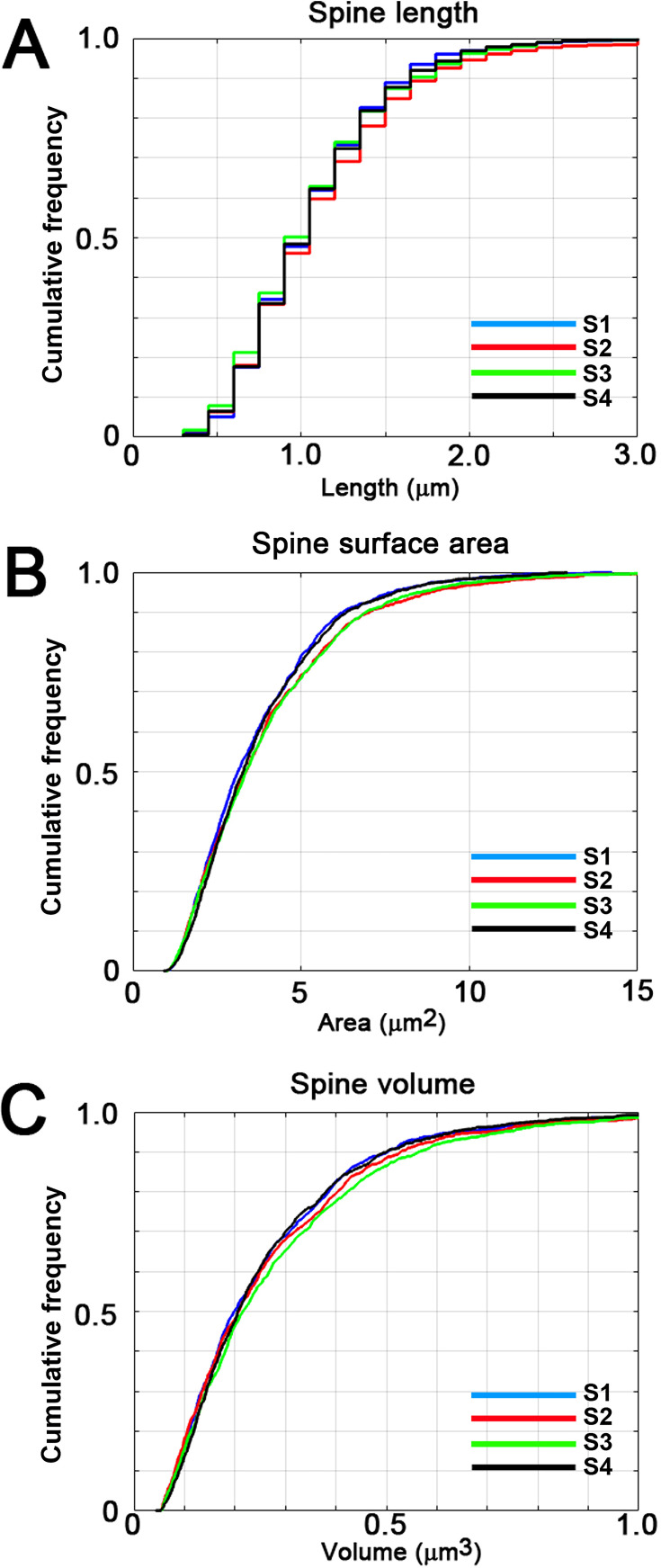
Cumulative frequency plots of spine length, surface area, and volume measured in four independent experiments performed >2 months apart. The cumulative frequency distributions of spine length (**A**), spine surface area (**B**), and spine volume (**C**) were obtained from wild-type data acquired by structured illumination microscopy (SIM) imaging across four mouse models: *Nlgn3*^R451C/(y or R451C)^ (S1), *Syngap1*^+/−^ (S2), *Pogz*^Q1038R/+^ (S3), and 15q11-13^dup/+^ (S4). The Kolmogorov–Smirnov test detected significant differences in only three of 18 possible pairwise comparisons: surface area of (S1) vs. (S3) (p = 0.017), volume of (S1) vs. (S3) (p = 0.032), and volume of (S3) vs. (S4) (p = 0.038).

**Figure 1—figure supplement 2.**
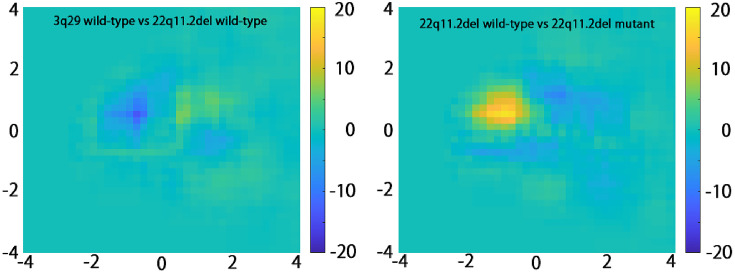
Subtracted density plots comparing wild-type cultures from 3q29^del/+^ and 22q11.2^del/+^ mice (left) and wild-type vs. mutant cultures from the 22q11.2^del/+^ line (right). The differences observed between independent wild-type culture preparations were substantially smaller than those observed between wild-type and mutant cultures, indicating that genotype-dependent effects exceeded inter-preparation variability.

Spine-shape parameters measured by conventional methods are difficult to compare directly when images are obtained from multiple independent experiments. This problem is caused by several factors, but the main factors are variability in image intensity, signal-to-noise ratio, and image aberration introduced by unoptimized imaging conditions. To test the stability of SIM-based spine imaging, we compared the spine size distribution across four samples prepared at time points separated by >2 months (Figure 1—figure supplement 1). The cumulative distribution curves of the four datasets showed extensive overlap, confirming the reproducibility of the SIM-based spine-shape measurements using independent culture samples.

Another technical issue in comparing spine shape is the heterogeneity of spine shapes and the absence of defined subclasses. Population-level spine analysis, therefore, requires a new strategy that enables objective comparisons of large numbers of spines with a continuum of morphologic features. We previously demonstrated that principal component analysis (PCA) was effective for comparing high-dimensional spine-shape features (descriptors) in a reduced-dimensional feature space (Kashiwagi et al., 2019). Indeed, a PCA-based dimensional reduction of a large number of spines, based on their descriptors, generated a two-dimensional plot of spine shape that closely matched conventional spine subtypes, such as thin, mushroom, and stubby (Figure 1E). To objectively compare the two spine datasets (control and disease model), we first generated plots of relative spine densities (number per area in feature space) mapped to 80 × 80 square blocks (Figure 1F), and then subtracted the corresponding plot pairs defined by genotype (control vs. mutant) or culture conditions. The resulting subtracted density plots contain comprehensive information about the differences in relative spine numbers with similar shape properties. As expected, there was some variation in the density plot patterns across experiments. However, subtracting the distributional data from wild-type and mutant samples effectively canceled out these differences. Moreover, the difference between the two wild-type samples was generally smaller than that between wild-type and mutant samples (Figure 1—figure supplement 2).

### Comparison and grouping of multiple mouse models based on morphological spine similarity

The subtracted density plots contain rich information about the population-level differences in spine shape between samples. Therefore, the generation and comparison of the plots for both ASD- and schizophrenia-associated mouse models will be useful for their unbiased grouping. We selected the following eight representative mouse models harboring either copy-number variation or single gene mutations associated with psychiatric disorders: male hemizygous or female homozygous mutant mice for neuroligin-3 R451C mutation (*Nlgn3*^R451C/(y or R451C)^) (Tabuchi et al., 2007; Etherton et al., 2011), heterozygous *Syngap1* mutant mice (*Syngap1*^+/−^) (Komiyama et al., 2002), heterozygous *Pogz* mutant mice (*Pogz*^Q1038R/+^) (Matsumura et al., 2020), mice paternal duplication of chromosome 7C (15q11-13^dup/+^) corresponding to human 15q11-13 duplication (Nakatani et al., 2009), mice with heterozygous deletion of chromosome 16B2,3 (3q29^del/+^) corresponding to human 3q29 deletion (Baba et al., 2019), mice heterozygous for deletion on chromosome 16qA13 (22q11.2^del/+^) corresponding to 1.5 Mb deletion on human 22q11.2 (Saito et al., 2021), mice heterozygous for *Setd1a* mutation (*Setd1a*^+/−^) (Nagahama et al., 2020), and Ca^2+^/calmodulin-dependent protein kinase IIα kinase-dead mutant mice (*Camk2a*^K42R/K42R^) (Yamagata et al., 2009). Previous studies on these mouse models confirmed the presence of multiple sensory, memory, and social endophenotypes of psychiatric disorders compared to controls. Of these, *Nlgn3*^R451C/(y or R451C)^, *Pogz*^Q1038R/+^, and 15q11-13^dup/+^ models mimic genetic variations found in ASD. Human mutations in the *SYNGAP1* gene are associated with intellectual disability and autistic behaviors. The 3q29^del/+^, 22q11.2^del/+^, and *Setd1a*^+/−^ models harbor genetic mutations associated with higher schizophrenia risk. CaMKII-related signaling pathway disruption has been implicated in the working memory deficits found in schizophrenia patients (Yamasaki et al., 2008; Featherstone et al., 2022). *CAMK2A* mutations in humans are linked to multiple mental disorders, including developmental disorders, ASD, and schizophrenia (Stephenson et al., 2017). The K42R mutation of *CAMK2A* does not correspond to any known human genetic variant, but the *CAMK2A* R8H mutation is linked to schizophrenia (Brown et al., 2021). Both R8H and K42R mutations in the N-terminal domain of CaMKIIα eliminate kinase activity; these mutations may have a similar impact on human mental disorders. Taken together, these mouse models cover a broad spectrum of human genetic aberrations associated with psychiatric disorders. Direct comparison of spine phenotypes could therefore provide important clues to underlying shared and disease-specific synaptic pathologies.

**Figure 2.**
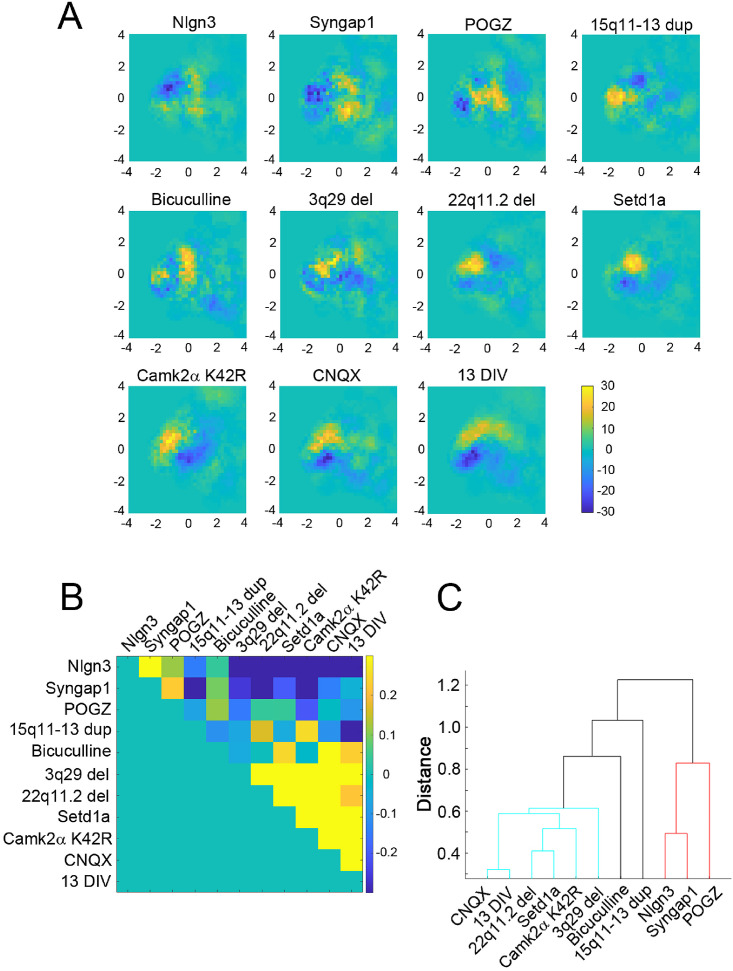
Spine population profiles for each model and corresponding control mouse line presented as subtracted density plots. (**A**) The subtracted density plots for eight disease mouse models (*Nlgn3*^R451C/(y or R451C)^, *Syngap1*^+/−^, *Pogz*^Q1038R/+^, 15q11-13^dup/+^, 3q29^del/+^, 22q11.2^del/+^, *Setd1a*^+/−^, and *Camk2a*^K42R/K42R^) and three different culture conditions (immature culture at 13 DIV, AMPA glutamate receptor blocker CNQX treatment, and GABA_A_ receptor blocker bicuculline treatment). The areas with a higher density of spines from mutant disease model mice are shown in yellow, and the areas with reduced density are shown in blue. The total number of spines (first number, *n*) analyzed from control and mutant mouse neurons and the corresponding number of dendrites (number in parentheses) are as follows: *Nlgn3*^R451C/(y or R451C)^; *n* = 1134 (58) and 1204 (59), *Syngap1*^+/−^; *n* = 991 (65) and 1371 (83), *Pogz*^Q1038R/+^; *n* = 1341 (72) and 1271 (72), 15q11-13^dup/+^; *n* = 1208 (68) and 1099 (63), 3q29^del/+^; *n* = 1429 (66) and 1408 (66), 22q11.2^del/+^; *n* = 1143 (66) and 914 (63), *Setd1a*^+/−^; *n* = 1381 (71) and 1405 (71), *Camk2a*^K42R/K42R^; *n* = 880 (58) and 763 (54). All data are from three independent culture preparations. (**B**) Matrix of the 2D cross-correlations among subtracted density plots. In the lower right area, a spine group showing similar morphological changes can be identified. This group corresponded to the mouse models of schizophrenia. (**C**) Unbiased clustering of spine samples showing two distinct groups corresponding to schizophrenia (cyan) and ASD (red).

For these comparisons, we prepared three independent cultures of embryonic hippocampi from each control–mutant pair and obtained 700–1500 3D-SIM spine images 3 weeks after plating for both mutant and control samples (*Nlgn3*^R451C/(y or R451C)^, *n* = 1134 and 1204; *Syngap1*^+/−^, *n* = 991 and 1371; *Pogz*^Q1038R/+^, *n* = 1341 and 1271; 15q11-13^dup/+^, *n* = 1208 and 1099; 3q29^del/+^, *n* = 1429 and 1408; 22q11.2^del/+^, *n* = 1143 and 914; *Setd1a*^+/−^, *n* = 1381 and 1405; *Camk2a*^K42R/K42R^; *n* = 880 and 763, control and mutant, respectively, Supplementary file 1). Initial comparison of the cumulative frequency plots for spine length, spine surface area, and spine volume alone did not provide sufficient information to infer their structural similarity (Figure 2—figure supplement 1), indicating that simple structural parameters are not effective in identifying disease-specific spine-shape characteristics. We next generated subtracted density plots from eight mouse models. These plots were variable, but there were certain similarities between the specific mutants (Figure 2A). We evaluated pairwise similarities by calculating the 2D cross-correlations of the subtracted density plots. The matrix of these 2D cross-correlation values yielded two major groups with high within-group similarity (Figure 2B), and this grouping was further confirmed by unbiased clustering analysis (Figure 2C). The ASD models *Nlgn3*^R451C/(y or R451C)^, *Syngap1*^+/−^, and *Pogz*^Q1038R/+^ were in the first group, while the second group contained the schizophrenia models 3q29^del/+^, 22q11.2^del/+^, and *Setd1a*^+/−^. This result is striking, as it indicates that information about spine shape alone can distinguish whether the culture preparation is derived from ASD- or schizophrenia-associated mouse models. It is also of note that there were greater similarities among the schizophrenia-associated mouse models than among the ASD-associated mouse models. This finding suggests convergence in spine pathophysiology across schizophrenia-associated mouse models.

**Figure 2—figure supplement 1.**
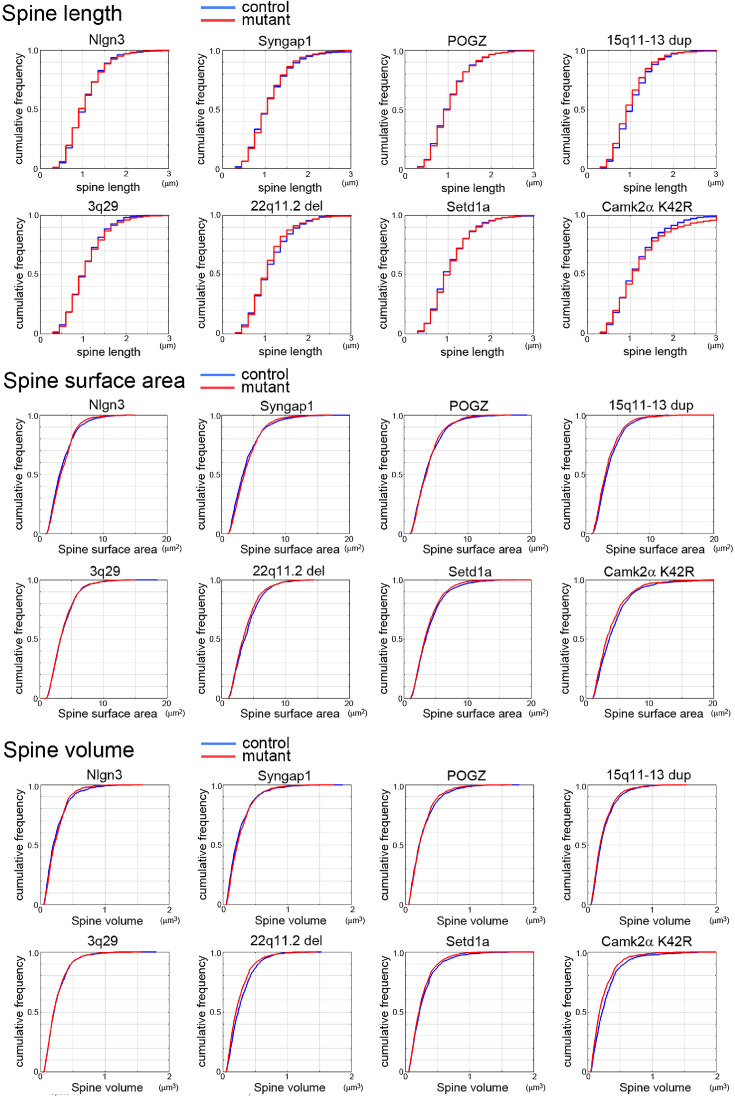
Cumulative frequency plots of spine length, surface area, and volume for the eight mouse mutants: *Nlgn3*^R451C/(y or R451C)^, *Syngap1*^+/−^, *Pogz*^Q1038R/+^, 15q11-13^dup/+^, 3q29^del/+^, 22q11.2^del/+^, *Setd1a*^+/−^, and *Camk2a*^K42R/K42R^.

In addition to the eight mouse models of psychiatric disorders, we generated subtracted density plots for three additional conditions: immature culture samples (13 days in vitro, DIV), treatment with the AMPA subtype glutamate receptor blocker CNQX, and treatment with the GABA_A_ receptor blocker bicuculline (Figure 2A). The subtracted density plots from the 13 DIV culture and the CNQX treatment were similar to those of schizophrenia-associated mouse models. This finding suggests that the hippocampal synapses in mouse models of schizophrenia are immature and inactive. In contrast, the subtracted density plot of the bicuculline-treated sample was distinct from that of both the schizophrenia and ASD-associated mouse models.

### Distinct spine shapes between ASD- and schizophrenia-associated mouse models

**Figure 3.**
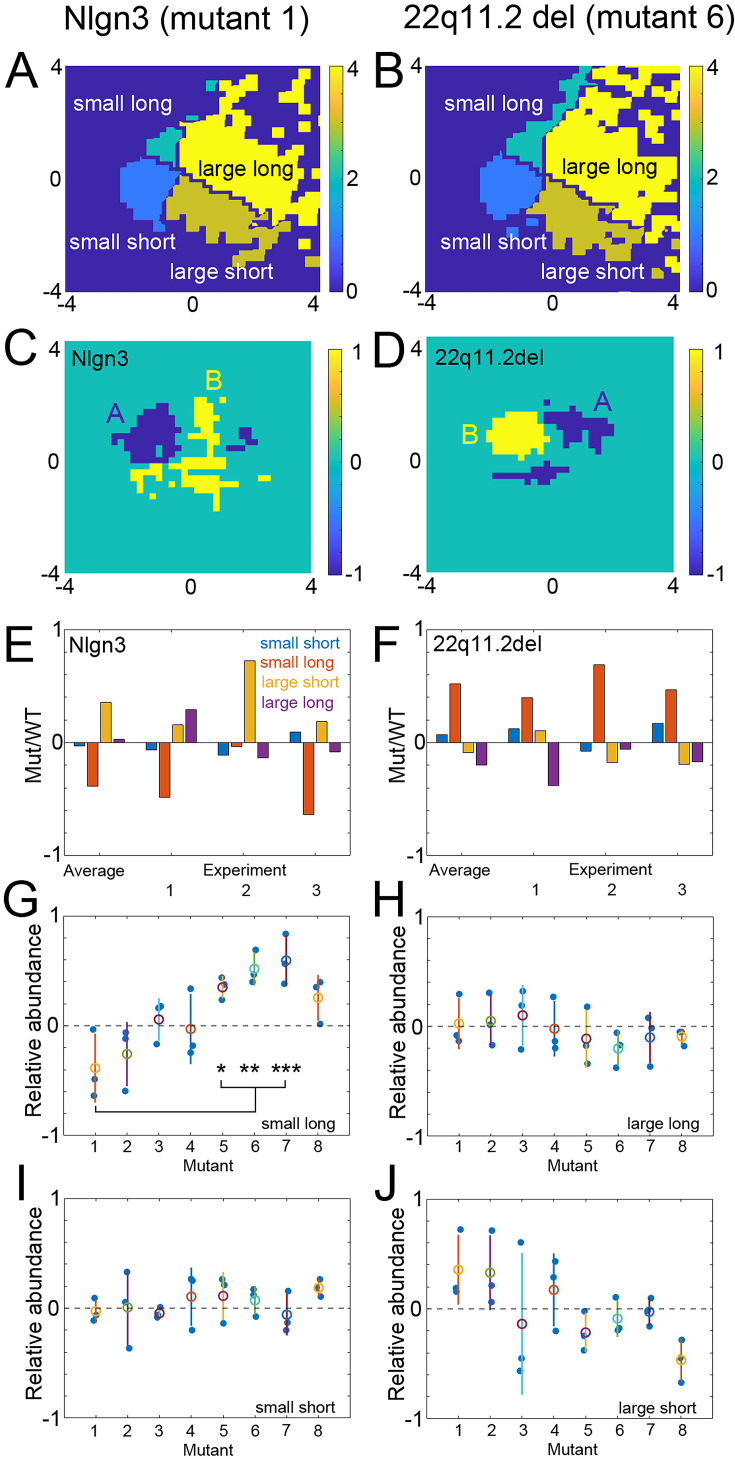
Distinct morphological properties of spines in cultured neurons derived from schizophrenia model mice compared with autism spectrum disorder (ASD) model mice. (**A, C, E**) Relative enrichment of four different subgroups of spines in *Nlgn3*^R451C/(y or R451C)^ neurons compared with wild-type neurons. In the projection plane of PC1 and PC2, four areas with distinct structural properties, namely (1) small and short, (2) small and long, (3) large and short, and (4) large and long, were defined (**A**). The map of areas with large differences (>3 × SD) between control and mutant mice in spine number per area in the feature space (**C**) indicates that the area enriched with wild-type spines (area B, yellow region) overlaps with the area of large spines. The area enriched with the mutant spines overlaps with the area of small spines (area A, blue region). The relative enrichment of mutant spines was summarized in (**E**). (**B, D, F**) Similar analysis of mutant spine enrichment in 22q11.2^del/+^ neurons. The map of spine distribution with four different properties (**B**), the map of areas enriched with wild-type or mutant spines (**D**), and the graph of relative enrichment of mutant spines (**F**) show that the mutant neurons were enriched with small and long spines. (**G–J**) Relative abundance of the four subgroups of spines in 8 mutant mouse models (1: *Nlgn3*^R451C/(y or R451C)^, 2: *Syngap1*^+/−^, 3: *Pogz*^Q1038R/+^, 4: 15q11-13^dup/+^, 5: 3q29^del/+^, 6: 22q11.2^del/+^, 7: *Setd1a*^+/−^, 8: *Camk2a*^K42R/K42R^). Three independent culture experiments with paired wild-type and mutant samples were performed. Group comparisons were performed using one-way ANOVA followed by Tukey’s post hoc test. (*p < 0.05, **p < 0.01, ***p < 0.005).

The subtracted density plots provide information on the structural properties of specific spine subsets that either increased or decreased in disease mouse models. We first defined the four areas within the PC1–PC2 plane with distinct shapes: (1) small and short, (2) small and long, (3) large and short, and (4) large and long (Figure 3A, B). We also identified the wild-type-enriched area (blue areas in Figure 3C, D) and the mutant-enriched area (yellow areas in Figure 3C, D) in the same projection plane. The comparison of these two types of areas suggests that larger spines are enriched in the *Nlgn3*^R451C/(y or R451C)^ mouse model, while small spines are enriched in the 22q11.2^del/+^ mouse model. Next, we calculated the normalized spine counts in the four areas for both wild-type and mutant samples and obtained the relative abundance (mutant/wild-type) for each area (Figure 3E, F). The average of three independent culture preparations confirmed the opposite trend between *Nlgn3*^R451C/(y or R451C)^ and 22q11.2^del/+^.

**Figure 3—figure supplement 1.**
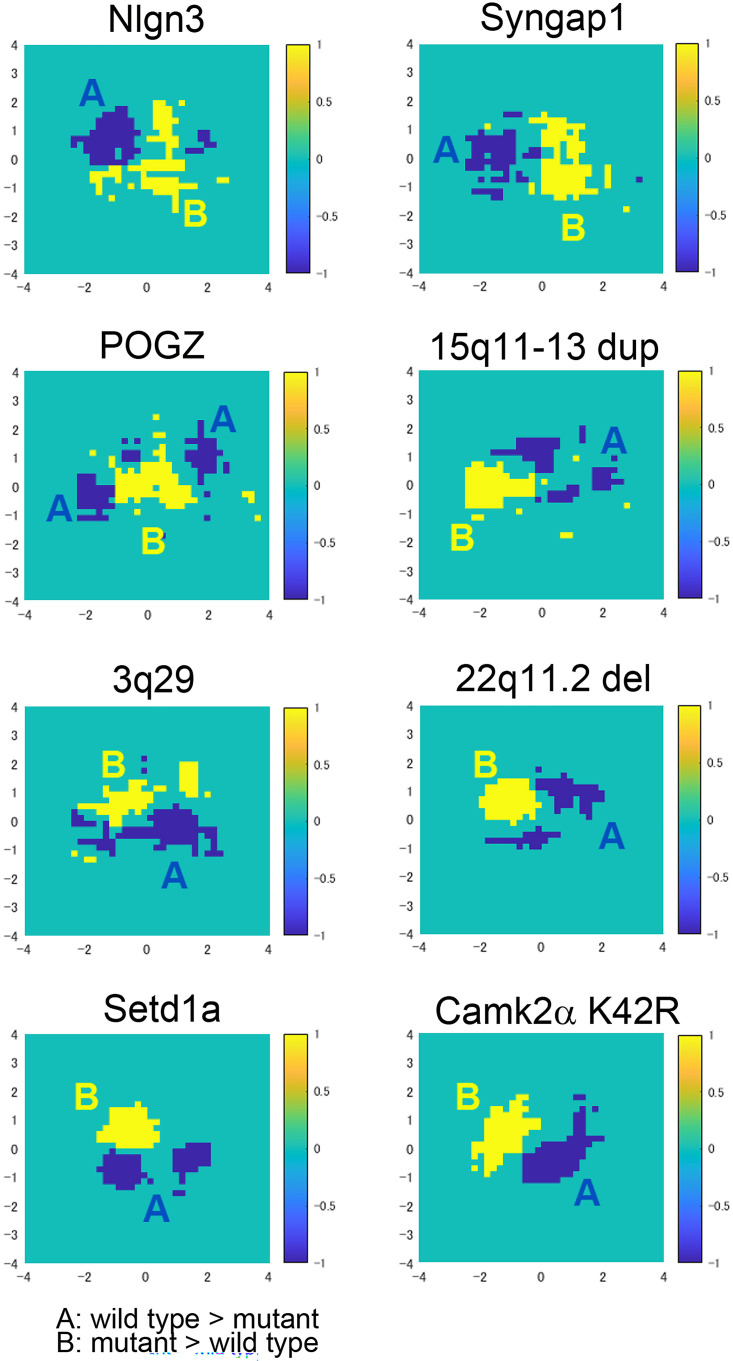
Areas where control (**A**: blue) or mutant (**B**: yellow) spines show a higher density within the feature space of the PC1–PC2 plane. The plots for all eight mouse models are presented. The differences in population-level spine properties between autism spectrum disorder (ASD)- and schizophrenia-associated mouse models were preserved within two groups. The areas enriched with mutant spines in *Nlgn3*^R451C/(y or R451C)^, *Syngap1*^+/−^, and *Pogz*^Q1038R/+^ models were on the right side of the plot, suggesting the abundance of large spines in this group. In contrast, the areas enriched with mutant spines in 3q29^del/+^, 22q11.2^del/+^, *Setd1a*^+/−^, and *Camk2a*^K42R/K42R^ models were positioned on the left side of the plot, indicating the abundance of small spines.

**Figure 3—figure supplement 2.**
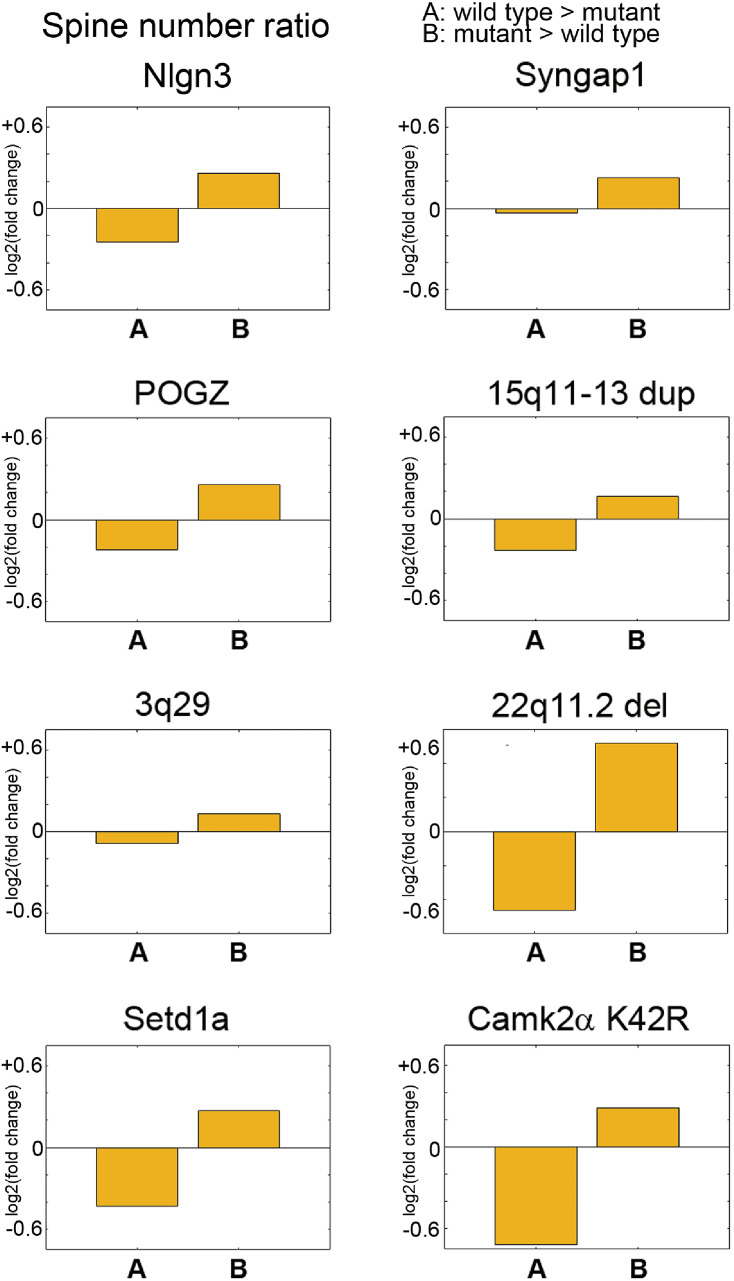
The relative numbers of spines within areas A and B in the feature space from Figure 3—figure supplement 1. The densities of mutant spines were higher in area B than in area A.

**Figure 3—figure supplement 3.**
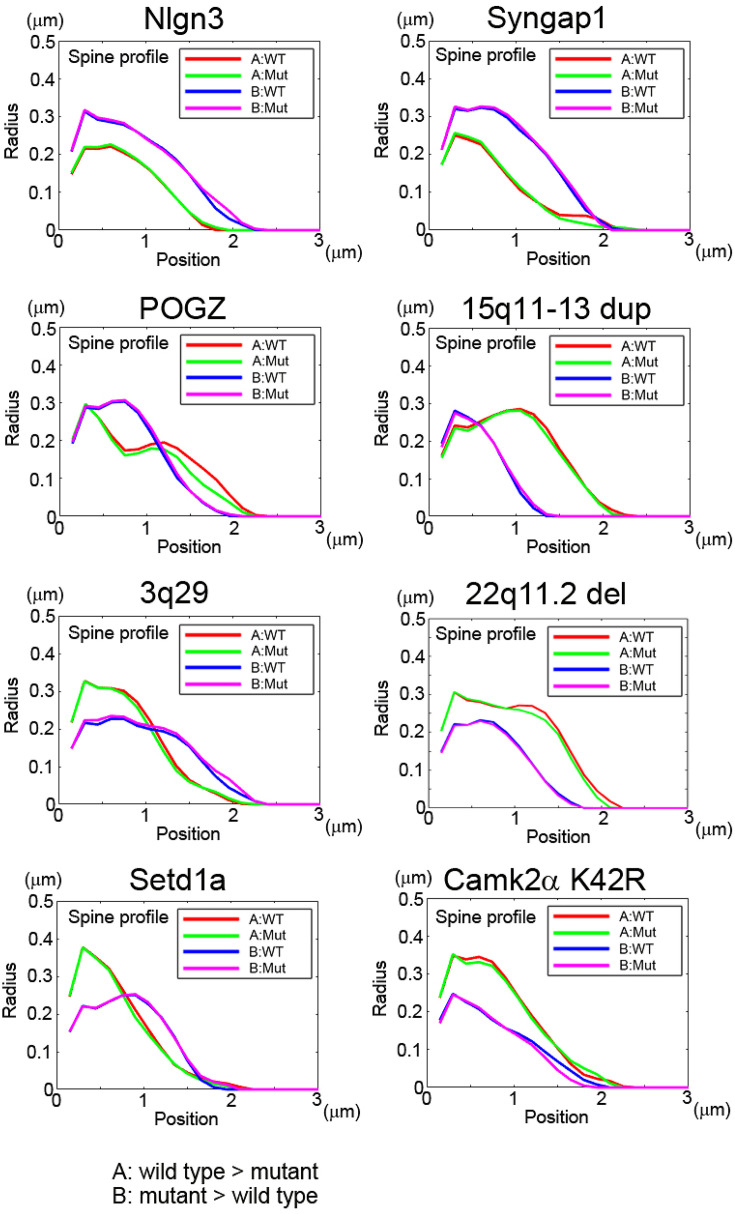
Profiles of different spine populations (spines in the control-dominant area A and the mutant-dominant area B for both wild-type and mutant neurons) were visualized by plotting the radius along the long axis. The numbers of control and mutant spines included in the analysis are as follows: *Nlgn3*^R451C/(y or R451C)^, *n* = 775 in area A and *n* = 513 in area B; *Syngap1*^+/−^, *n* = 668 in area A and *n* = 500 in area B; *Pogz*^Q1038R/+^, *n* = 580 in area A and *n* = 815 in area B; 15q11-13^dup/+^, *n* = 378 in area A and *n* = 797 in area B; 3q29^del/+^, *n* = 627 in area A and *n* = 824 in area B; 22q11.2^del/+^, *n* = 308 in area A and *n* = 575 in area B; *Setd1a*^+/−^, *n* = 472 in area A and *n* = 808 in area B; *Camk2a*^K42R/K42R^, *n* = 237 in area A and *n* = 634 in area B.

Figure 3G–J summarizes the relative abundance of mutant spines in four areas with distinct spine shapes. We found that the relative abundance of mutant spines in area 2 (small and long spines) differed significantly between genotypes (ANOVA, p < 0.001). Group comparisons by Tukey’s post hoc test indicate significant differences between *Nlgn3*^R451C/(y or R451C)^ mutant and three schizophrenia-related mutants: 3q29^del/+^, 22q11.2^del/+^, and *Setd1a*^+/−^ (Figure 3G). The opposite trend between areas 2 (small and long spines) and 3 (large and short spines) further confirms distinct spine-shape properties in ASD- and schizophrenia-related mouse models. The areas within the subtracted density plots with enriched control or mutant spines illustrate the characteristics of spine populations across mouse models (Figure 3—figure supplements 1 and 2). The shape profiles of spines confirmed smaller spine volume of schizophrenia-related mouse models (Figure 3—figure supplement 3).

### Altered initial growth of dendritic spines in mouse models of schizophrenia

**Figure 4.**
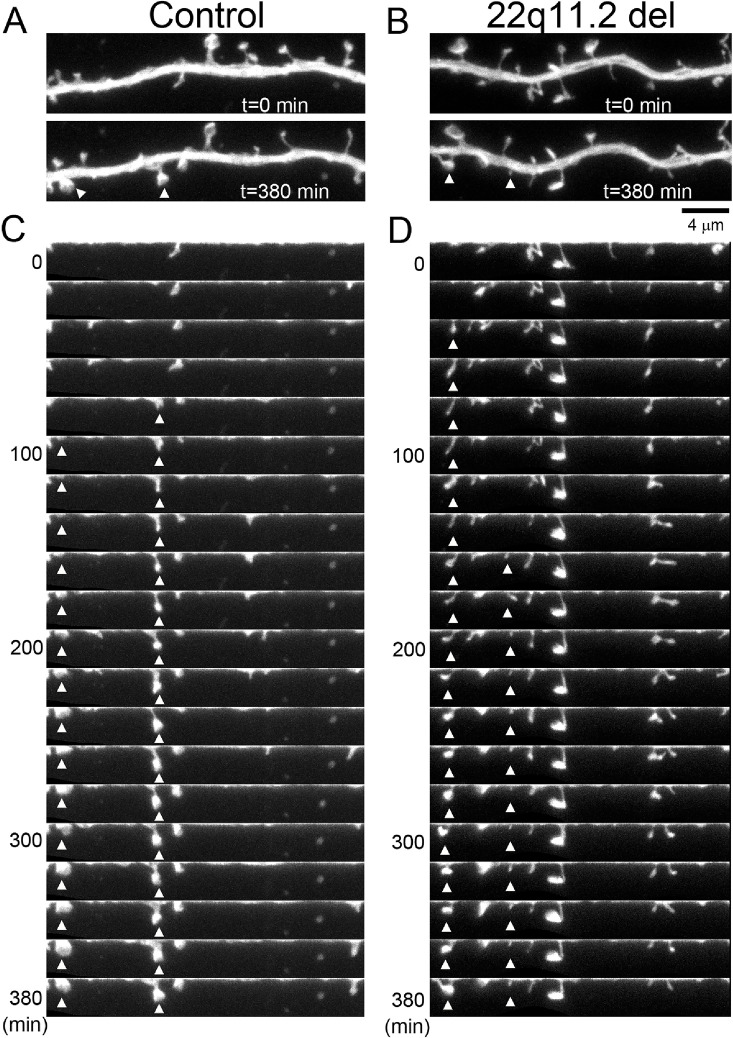
Time-lapse imaging of neurons derived from 22q11.2^del/+^ mice and corresponding control mice. Images of dendritic segments from control neurons (**A**) and 22q11.2^del/+^ neurons (**B**) at two different time points. Montages of time-lapse images from control neurons (**C**) and 22q11.2^del/+^ neurons (**D**). The curved dendrites were straightened, revealing newly formed spines (arrowheads) as fluorescent objects appearing at the edge of the dendritic shafts. Bar, 4 μm.

The subtracted density plots reveal common changes in the spine population across schizophrenia-associated mouse models. The mutant-spine-enriched areas also contain small, elongated spines, suggesting dysfunction in either the generation of nascent spines or the shrinkage of existing spines. To examine potential abnormalities in spine dynamics, we performed time-lapse imaging of hippocampal neuron cultures derived from two schizophrenia-associated mouse models, 22q11.2^del/+^ and *Setd1a*^+/−^, and the *Nlgn3*^R451C/(y or R451C)^ mouse model as a reference (Figure 4).

**Figure 5.**
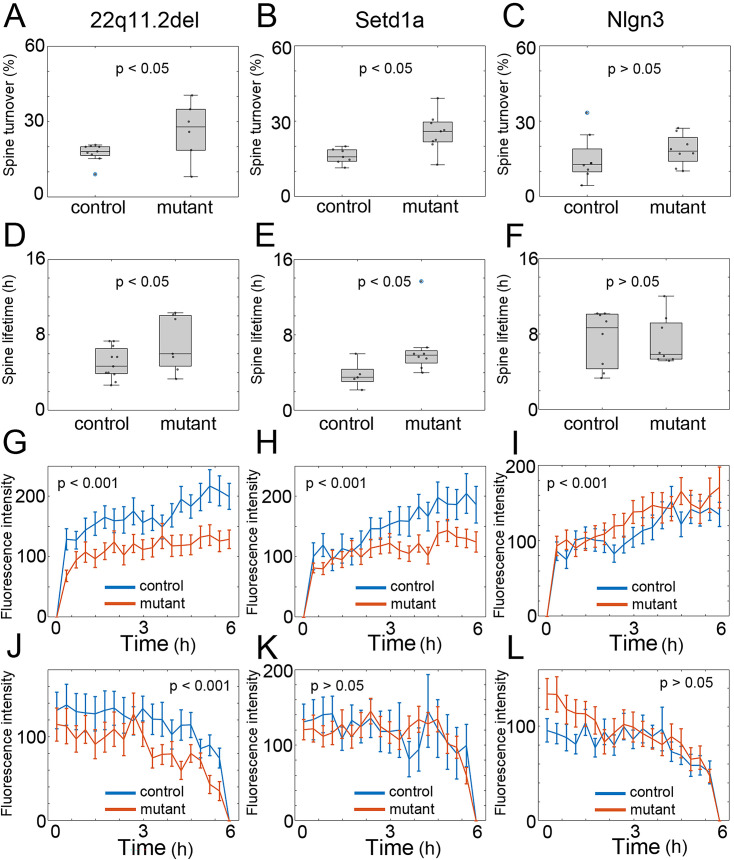
Turnover rate, lifetime, and growth/shrinkage profiles of dendritic spines in cultured neurons derived from 22q11.2^del/+^, *Setd1a*^+/−^, and *Nlgn3*^R451C/(y or R451C)^ mice, as well as the corresponding controls. (**A–C**) Spine turnover rates in the three mutant mouse models compared to the controls. Spine turnover rates were analyzed using a linear mixed-effects model with genotype as a fixed effect and plate, cell, and dendrite as nested random effects, showing a significant effect of genotype only in 22q11.2^del/+^ and *Setd1a*^+/−^ mice (*n* = 16 dendrites/9 cells/5 plates in control and 11 dendrites/6 cells/4 plates in 22q11.2^del/+^, *n* = 10 dendrites/7 cells/4 plates in control and *n* = 14 dendrites/9 cells/6 plates in *Setd1a*^+/−^, *n* = 8 dendrites/8 cells/3 plates in control and *n* = 8 dendrites/8 cells/3 plates in *Nlgn3*^R451C/(y or R451C)^). (**D–F**) Lifetimes of transient spines for the three mutant mouse models compared with corresponding controls. Spine lifetime was analyzed using a linear mixed-effects model accounting for the hierarchical structure of the data (spines nested within dendrites, cells, and culture plates). The analysis revealed a significant effect of genotype only in 22q11.2^del/+^ and *Setd1a*^+/−^ neurons (*n* = 186 spines/11 dendrites/7 cells/5 plates in control and *n* = 166 spines/7 dendrites/4 cells/4 plates in 22q11.2^del/+^, *n* = 82 spines/5 dendrites/5 cells/4 plates in control and *n* = 202 spines/8 dendrites/8 cells/5 plates in *Setd1a*^+/−^, *n* = 98 spines/8 dendrites/8 cells/3 plates in control and *n* = 125 spines/8 dendrites/8 cells/3 plates in *Nlgn3*^R451C/(y or R451C)^). (**G–I**) Temporal patterns of spine growth (*n* = 60 spines/11 neurons/7 cells/5 plates in control and *n* = 50 spines/7 dendrites/4 cells/4 plates in 22q11.2^del/+^, *n* = 25 spines/5 dendrites/5 cells/4 plates in control and *n* = 65 spines/8 dendrites/8 cells/5 plates in *Setd1a*^+/−^, *n* = 43 spines/8 dendrites/8 cells/3 plates in control and *n* = 41 spines/7 dendrites/7 cells/2 plates in *Nlgn3*^R451C/(y or R451C)^). Spine volume trajectories were analyzed using linear mixed-effects models incorporating nested random effects (spine within dendrite within cell within culture plate) to account for the hierarchical structure of the data. Newly formed spines in both 22q11.2^del/+^ and *Setd1a*^+/−^ neurons were significantly smaller than those in wild-type neurons. In contrast, newly formed spines in *Nlgn3*^R451C/(y or R451C)^ neurons were significantly larger than those in wild-type neurons. (**J–L**) Temporal patterns of spine shrinkage (*n* = 39 spines/10 dendrites/6 cells/5 plates in control and *n* = 37 spines/7 dendrites/4 cells/4 plates in 22q11.2^del/+^, *n* = 15 spines/5 dendrites/5 cells/4 plates in control and *n* = 55 spines/8 dendrites/8 cells/5 plates in *Setd1a*^+/−^, *n* = 28 spines/8 dendrites/8 cells/3 plates in control and *n* = 35 spines/8 dendrites/8 cells/3 plates in *Nlgn3*^R451C/(y or R451C)^ neurons). Spine volume trajectories were analyzed using linear mixed-effects models as in (**G–I**). In the 22q11.2^del/+^ neurons, spines undergoing elimination were significantly smaller than those in wild-type neurons. This effect was not detected in *Setd1a*^+/−^ and *Nlgn3*^R451C/(y or R451C)^ neurons.

A linear mixed-effects model indicated significant effects of genotype in the increase of spine turnover rate in both 22q11.2^del/+^ and *Setd1a*^+/−^ models (*F*(1,25) = 5.79, p = 0.024 for 22q11.2^del/+^ and *F*(1,22) = 7.33, p = 0.013 for *Setd1a*^+/−^). In contrast, no significant effects were observed in the *Nlgn3*^R451C/(y or R451C)^ model (Figure 5A–C). There was a significant effect of genotype in both 22q11.2^del/+^ and *Setd1a*^+/−^ models also for the prolonged lifetime of transient spines (a linear mixed-effects model: 22q11.2^del/+^; *F*(1,336) = 5.33, p = 0.022, *Setd1a*^+/−^; *F*(1,282) = 6.38, p = 0.012), with no significant difference in *Nlgn3*^R451C/(y or R451C)^ model (Figure 5D–F).

We further compared the initial increase in spine volume between genotypes (Figure 5G–I). Linear mixed-effects models incorporating nested random effects revealed significantly smaller initial spine volumes in both 22q11.2^del/+^ and *Setd1a*^+/−^ models (genotype effect: p < 0.001 for 22q11.2^del/+^ and p < 10^−^⁷ for *Setd1a*^+/−^). The spines in both mutants also displayed a significant reduction in spine volume increase (p < 0.001). In contrast, newly formed spines in the *Nlgn3*^R451C/(y or R451C)^ neurons were significantly larger than those in wild-type neurons (p < 10⁻⁴) with preserved time-course of spine growth.

Similar analyses of spine volume reduction before spine loss using linear mixed-effects models identified significantly smaller initial spine size in the 22q11.2^del/+^ model (p < 10^−6^), while no significant differences in the initial spine volume were found for the *Setd1a*^+/−^ model (Figure 5J–L). The temporal trajectories of spine shrinkage before their loss were not significantly altered in both 22q11.2^del/+^ and *Setd1a*^+/−^ models. The *Nlgn3*^R451C/(y or R451C)^ model showed a significantly different time-course of spine shrinkage (p < 0.05), while the initial spine size was not altered. Taken together, these results suggest that the smaller size of the initial spine, slower growth of nascent spines, and enhanced spine turnover contribute to the development of schizophrenia-related spine phenotypes.

**Figure 6.**
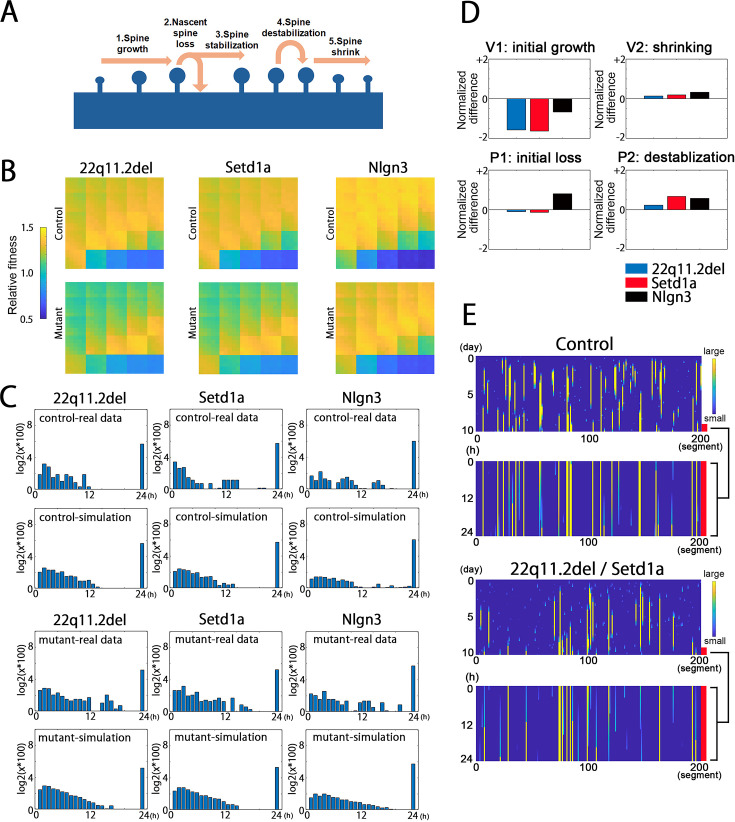
Simulations of long-term spine turnover. (**A**) The model of spine dynamics. Five successive phases of spine state transitions were defined. Phase 1: newly formed spines grow at speed V1. Phase 2: nascent spines are eliminated with probability P1. Phase 3: nascent spines are stabilized when volume reaches an upper threshold. Phase 4: stable spines are destabilized with probability P2. Phase 5: spines shrinking at a rate V2 are lost after reaching a lower threshold. (**B**) Pseudocolor maps of 625 different combinations of these parameters to identify those best fitting the experimental data. The color-coded values indicate the fitness of the simulation results to both the spine lifetime distribution profile and the turnover rate. (**C**) Frequency histograms of spine lifetimes for the three mouse models and controls (experimental data), and the results of simulations. The bin with a lifetime of 24 hr corresponds to the spines that persisted throughout the imaging period. (**D**) Differences in V1, V2, P1, and P2 between mutant mice and controls, expressed as differences in the four parameters normalized by the control condition values. (**E**) Plots of individual spine turnover simulated using parameters that best fit the experimental data from control and schizophrenia-associated mouse models. The upper plots show the progression of spine formation along 200 dendritic segments (50 μm) over 10 days. The lower plots show the enlargement of the last 24 hr of spine turnover. Color indicates spine size.

To further examine the relationship between the spine growth rate and lifetime, we performed simulations using the computational model shown in Figure 6A. In this model, new spines appear randomly along the dendritic shaft according to a density-dependent probability function, then increase in volume with speed V1 (phase 1), are eliminated with probability P1 (phase 2), and are converted to stable spines upon reaching a volume threshold (phase 3). These stable spines are subsequently destabilized with low probability P2 (phase 4) and start to shrink with speed V2 (phase 5). When these shrinking spines reach a threshold, they are permanently removed. We did not incorporate the initial spine size as a model parameter because of its large variability, which made parameter optimization difficult. This model was run over 10 days of neuronal differentiation in culture (corresponding to the period of spine density increase from 8 to 17 DIV) with 625 different combinations of V1, V2, P1, and P2 to identify the combination with the best fit to the experimental data for spine turnover of 15–18 DIV neurons derived from mutants 22q11.2^del/+^, *Setd1a*^+/−^, and *Nlgn3*^R451C/(y or R451C)^ over 24 hr (Figure 6B, Figure 6—figure supplement 1A). The best-fitted models all yielded spine lifespan distributions matching observations of the three mouse mutants (Figure 6C). The two characteristics of the spines in 22q11.2^del/+^ and *Setd1a*^+/−^ were also preserved in the best-fitted model. First, spine density in the schizophrenia-associated mouse models was lower in both the simulation and experimental data (simulation: 0.57 ± 0.029 and 0.40 ± 0.027/μm for the control and the best-fitted for schizophrenia models, shown in Figure 6—figure supplement 1B, experiment; 0.69 ± 0.17 and 0.56 ± 0.20/μm for the control and 22q11.2^del/+^, 0.70 ± 0.15 and 0.64±0.16/μm for the control and *Setd1a*^+/−^). Second, the size of transient spines was smaller in schizophrenia-associated mouse models in the simulation (0.33 ± 0.033and 0.20 ± 0.024 (a.u.) for the control and the best-fitted schizophrenia, Figure 6—figure supplement 1C), consistent with data on spine nanostructure (Figure 3G, J).

**Figure 6—figure supplement 1.**
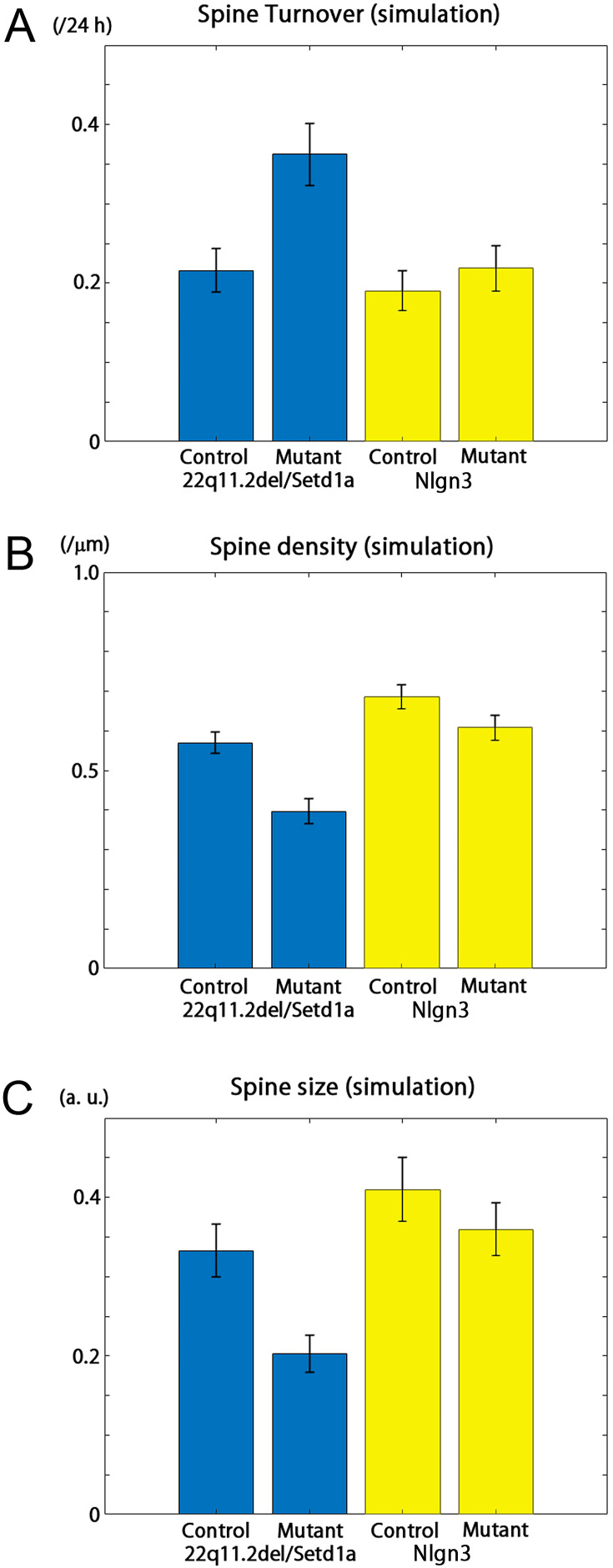
Spine turnover (**A**), density (**B**), and size (**C**) from simulation data. Simulation parameters were tuned to fit experimental results from control and mutant models. Means and standard deviations are shown.

After the selection of the best-fitted parameters for three mouse models, we compared the optimal values of V1, V2, P1, and P2 between mouse models (Figure 6D). The nascent spine volume increase (V1) was slower in schizophrenia models than in corresponding controls. In turn, the probability of nascent spine loss (P1) was higher in the ASD model. In all three disease models, the probability of spine destabilization (P2) was moderately higher than that in the corresponding controls. Additional simulation results with different P2 values indicate that P2 has a small effect on the overall pattern of spine dynamics. These results suggest that a slow spine volume increase in schizophrenia model mice results in the accumulation of nascent spines with smaller volumes. On the other hand, the spines of ASD model mice grow at a rate similar to that of control spines, but are more likely to be eliminated both in the nascent stage and after maturation.

Simulated trajectory plots of individual spines along the dendritic shaft, using the best-fit parameters, also reflected the differential patterns of spine turnover in culture preparations derived from control and schizophrenia model mice (Figure 6E). In culture preparations derived from schizophrenia-associated mouse models, there were fewer spines with a lifetime longer than 2 or 3 days, while there was a greater number of spines formed and eliminated within 24 hr. This increase in the number of less stable spines explains the pattern of spine lifetimes shown in Figure 5D, E. In summary, these simulations suggest that a reduced spine maturation rate can account for three key properties of schizophrenia-related spines: the greater number of small-volume spines, enhanced turnover, and reduced spine density.

### Screening of molecules responsible for altered spine properties in schizophrenia-associated mouse models

Both SIM-based imaging and time-lapse imaging suggest that the initial growth phase of spines is slower in schizophrenia model mice. To identify candidate genes that may contribute to this growth impairment, we performed RNA sequencing of libraries derived from the cultured neurons of eight psychiatric disorder mouse models and corresponding controls, and filtered the results for differentially expressed genes (DEGs) (Supplementary files 2 and 3). The DEGs present in three or more schizophrenia-related mouse models are listed in Figure 7—figure supplement 1. In the hippocampi of schizophrenia model mice, the expression levels of *Met*, *Osr1*, *Arhgap15*, and *Rnf170-ps* were downregulated, whereas those of *Cip4*, *Ecrg4*, and *Npas4* were upregulated. The majority of these DEGs have been previously implicated in the pathology of schizophrenia (*Met*) (Qiu et al., 2014), synaptic plasticity (*Npas4*) (Bloodgood et al., 2013; Spiegel et al., 2014), or signaling pathways in the nervous system (*Arhgap15*, Zamboni et al., 2016; *Cip4*, Ba et al., 2016; and *Ecrg4*, Nakatani et al., 2019).

**Figure 7.**
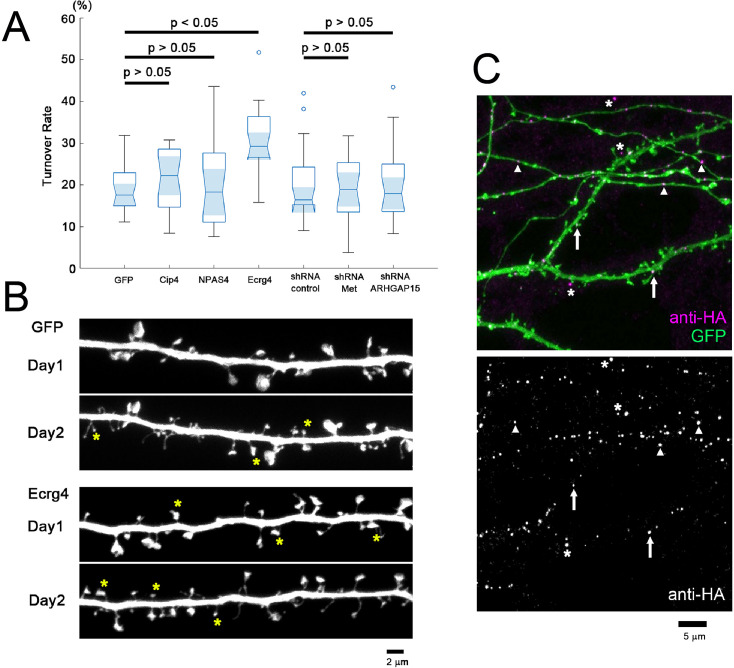
Manipulation of candidate gene expression in wild-type hippocampal neurons and its effects on spine turnover. (**A**) Turnover rates of dendritic spines in neurons transfected with an overexpression plasmid encoding *Cip*4, *Npas4*, or *Ecrg4*, or a plasmid encoding an shRNA targeting *Met* or *Arhgap15*, together with a green fluorescent protein (GFP) expression plasmid. Spine turnover rates were calculated from the images of GFP-expressing dendrites taken at an interval of 24 hr. Among the five differentially expressed genes (DEGs), only the upregulation of *Ecrg4* selectively increased the spine turnover rate. Linear mixed-effects modeling with experiment as a random factor revealed a significant effect of overexpression treatment (*F*(3,8) = 4.59, p = 0.038), driven by *Ecrg4* (p = 0.013), whereas shRNA manipulations showed no significant effect (*F*(2,6) = 0.29, p = 0.76). (results from *n* = 22 dendrites/11 neurons/3 culture plates derived from independent primary culture for all conditions except the *Met* shRNA groups, where *n* = 21 dendrites from 11 neurons were included in the analysis). (**B**) Fluorescence images of dendritic segments expressing GFP or GFP plus Ecrg4 on days 1 and 2. Newly formed spines are marked by asterisks. Bar, 2 μm. (**C**) Images of dendrites and axons expressing HA-tagged Ecrg4 together with GFP. Anti-HA immunocytochemistry revealed the presence of immunopositive puncta both in the dendrites (arrows) and axons (arrowheads). Some clusters could be detected in the extracellular space (asterisks). The upper image is the overlay of the anti-HA signal (magenta) and GFP (green). The lower image shows the distribution of the anti-HA signals. Bar, 5 μm.

If these genes do regulate spines in schizophrenia-associated mouse models, manipulation of these genes in wild-type neurons may induce a spine phenotype similar to that observed in these models. To test this possibility, we downregulated *Met* and *Arhgap15*, and upregulated *Cip4*, *Ecrg4*, and *Npas4* in separate wild-type hippocampal neuron cultures and measured spine dynamics using time-lapse imaging (Figure 7A, Figure 7—figure supplement 2). The application of a linear mixed-effects model to knockdown experiments revealed no significant effect of treatment (*F*(2,6) = 0.29, p = 0.76). In the overexpression experiment, the analysis using a linear mixed-effects model showed a significant main effect of treatment (*F*(3,8) = 4.59, p = 0.038), with a significant increase in spine turnover rate by *Ecrg4* overexpression (*β* = 0.49 ± 0.16SE, *t*(8) = 3.16, p = 0.013). Thus, the aberrant expression of *Ecrg4* may be related to the spine phenotype of schizophrenia model mice.

**Figure 7—figure supplement 1.**
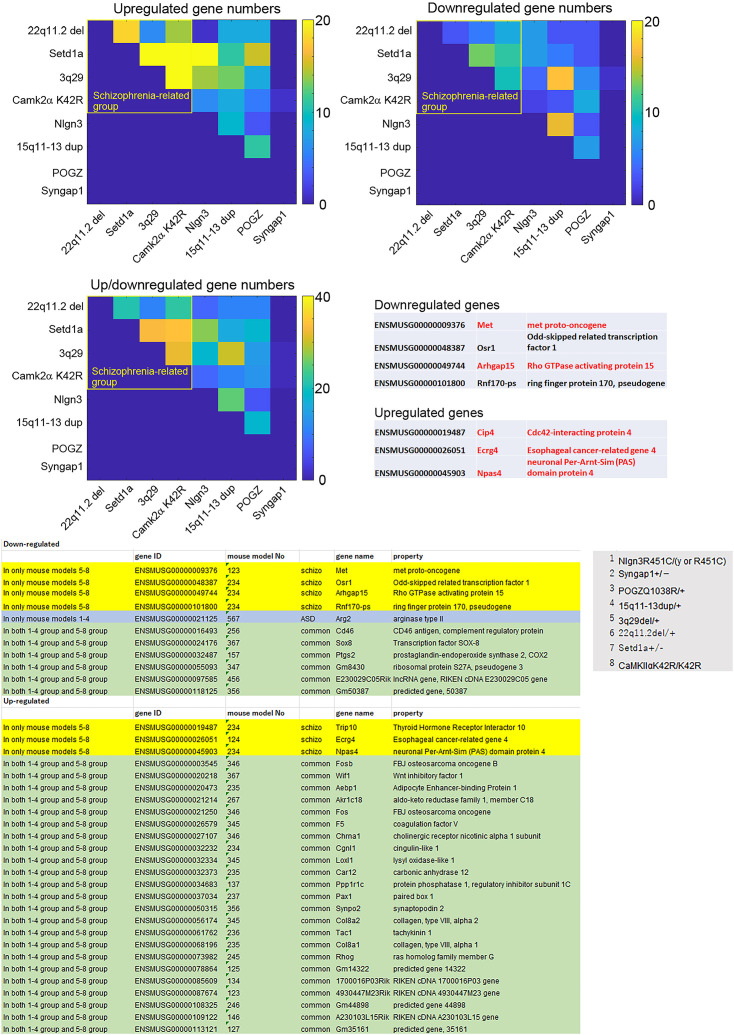
Pseudocolor presentation of differentially expressed genes (DEGs) between mutant and corresponding control mice. The number of shared DEGs was higher in the schizophrenia-related mouse models than in the autism spectrum disorder (ASD)-related mouse models. Mouse gene identifiers (ENSMUSG) and gene names for DEGs shared by three or four schizophrenia-associated mouse models and not differentially expressed in ASD-associated mouse models are presented. DEGs analyzed for their effects on spines are shown in red. Color indicates the number of DEGs. The lower table shows all DEGs shared by three or four mouse models, irrespective of their grouping as ASD- or schizophrenia-related.

**Figure 7—figure supplement 2.**
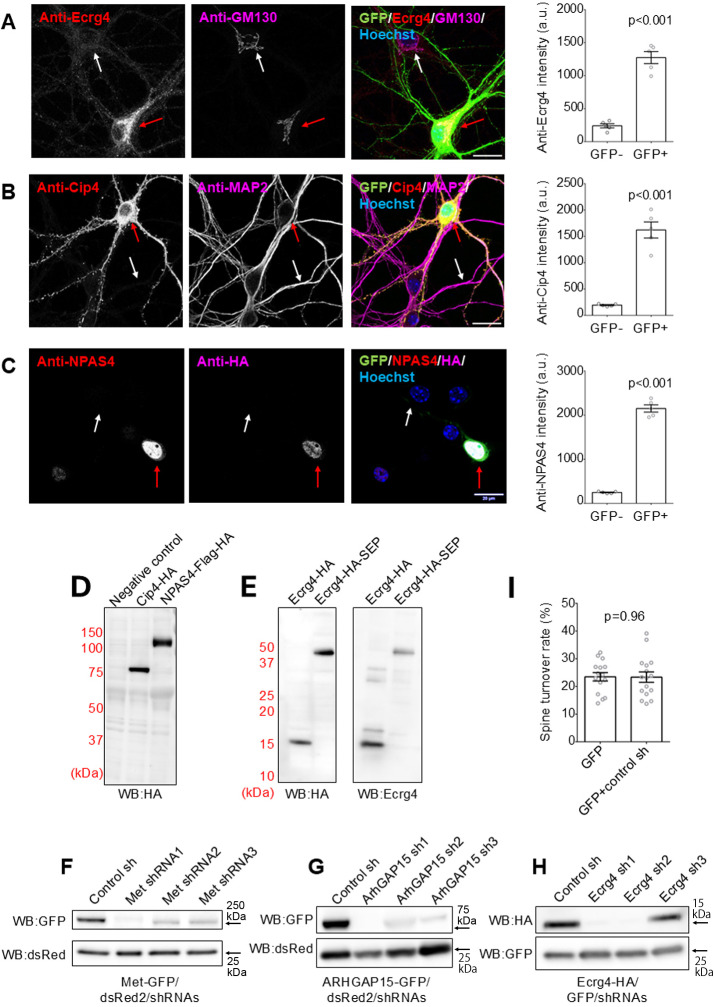
Validation of overexpression and shRNA knockdown constructs for DEGs identified in schizophrenia-associated mouse models. (**A–C**) Validation of overexpressed tagged constructs by immunocytochemistry in hippocampal neurons. Bar = 20 μm. The data are presented as mean ± SEM (*N* = 5 neurons each). Red allows; transfected neurons, white arrows; non-transfected neurons. (**A**) Immunocytochemistry of hippocampal neurons expressing Ecrg4-HA with green fluorescent protein (GFP) using anti-Ecrg4 antibody. Anti-GM130 antibody staining detected the Golgi apparatus. (**B**) Immunocytochemistry of hippocampal neurons expressing Cip4-HA with GFP using anti-Cip4 antibody. Anti-MAP2 antibody detected neuronal dendrites. (**C**) Anti-NPAS4 and anti-HA immunocytochemistry of hippocampal neurons expressing NPAS4-Flag-HA with GFP. (**D, E**) Immunoblot verification of tagged construct expression in COS-7 cells. (**D**) Immunoblotting in total cell lysate from COS-7 cells expressing Cip4-HA or NPAS4-Flag-HA. The estimated molecular weights of Cip4-HA and NPAS4-Flag-HA are 64 and 89 kDa, respectively. (**E**) Immunoblotting using both anti-HA and anti-Ecrg4 in total cell lysate from COS-7 cells expressing Ecrg4-HA or Ecrg4-HA-SEP. The estimated molecular weights of Ecrg4, Ecrg4-HA, and Ecrg4-HA-SEP are 17, 18, and 45 kDa, respectively. (**F–H**) Verification of knockdown effects of shRNAs by immunoblotting of COS-7 cells. Immunoblotting in total cell lysates from COS-7 cells transfected with the expression vectors of either *Met*-GFP (**F**), *ArhGAP15*-GFP (**G**), or Ecrg4-HA (**H**), together with dsRed2 and shRNAs targeting the corresponding transcripts. (**I**) Measurement of spine turnover rate in neurons transfected with a control shRNA construct together with GFP expression vectors. For the experimental control, we used neurons transfected only with GFP. The data are presented as mean ± SEM (*n* = 15 dendrites from 9 neurons for GFP control, *n* = 16 dendrites from 8 neurons for GFP plus control shRNA). Figure 7—figure supplement 2—source data 1.PDF file containing original western blots for Figure 7—figure supplement 2, indicating the relevant bands and molecular weight standards. Figure 7—figure supplement 2—source data 2.Original files for western blot analysis displayed in Figure 7—figure supplement 2.

**Figure 7—figure supplement 3.**
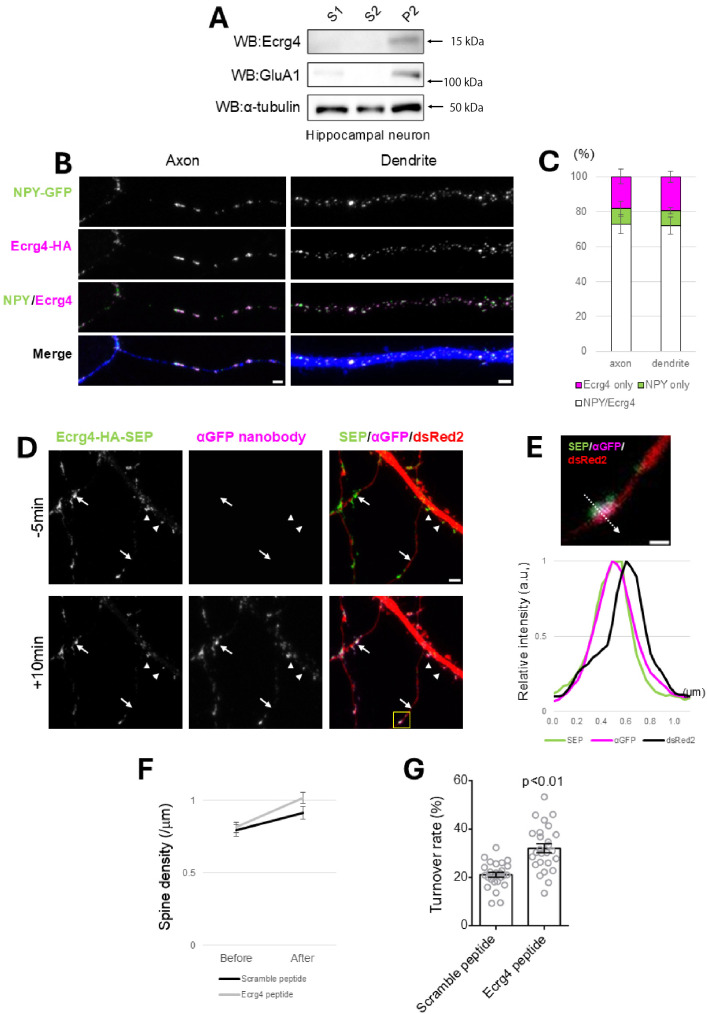
Expression and distribution of Ecrg4 in cultured hippocampal neurons. (**A**) Preferential localization of Ecrg4 protein in the membrane fraction. Immunoblotting of Ecrg4, GluA1 AMPA-type glutamate receptor, and α-tubulin in the remaining fraction after removal of nuclei (S1), supernatant after centrifugation (S2), and resulting pellet (P2). (**B**) Images of an immunostained hippocampal neuron expressing HA-tagged Ecrg4 and NPY-GFP. The fluorescence signals were partially colocalized, suggesting Ecrg4 protein accumulation in dense-core vesicles. Bar = 2 μm. (**C**) Quantification of the overlap between puncta immunopositive for HA-tagged Ecrg4 and NPY-GFP. *N* = 4 cells from 1 dish. (**D**) Surface labeling with the anti-GFP nanobody revealing clusters of SEP-tagged Ecrg4 in dsRed2-positive axons (arrows) and dendrites (arrowheads). A nanobody was applied at time *t* = 0 min. Bar = 2 μm. (**E**) Enlarged image of a single Ecrg4 puncta in the axon, marked by a yellow square in (**D**), with the fluorescence intensity profile along the dashed arrow. Bar = 0.5 μm. (**F**) The spine density of cultured hippocampal neurons 24 hr after administration of either Ecrg4 peptides or scrambled peptides (*n* = 26 dendrites and 13 neurons for each condition). (**G**) Spine turnover rate of neurons treated with Ecrg4 peptides or scrambled peptides (*n* = 26 dendrites and 13 neurons for each condition). Figure 7—figure supplement 3—source data 1.PDF file containing original western blots for Figure 7—figure supplement 3, indicating the relevant bands and molecular weight standards. Figure 7—figure supplement 3—source data 2.Original files for western blot analysis displayed in Figure 7—figure supplement 3.

*Ecrg4* encodes a precursor protein that yields several short hormone-like peptides via multiple cleavage sites (Richter et al., 2023). Ecrg4 has been reported to function in cell proliferation, inflammation, and tumor suppression. However, there is limited information on the neuronal functions of Ecrg4. Therefore, we further analyzed the cellular distribution of Ecrg4 in wild-type hippocampal neurons to assess its potential synaptic functions. The Ecrg4 protein was detected in the membrane fraction of cultured hippocampal neurons (Figure 7—figure supplement 3A). In addition, HA-tagged Ecrg4 showed punctate localization patterns within the neuronal cytoplasm (Figure 7C). A fraction of HA-tagged Ecrg4 puncta localized at axonal varicosities (arrowheads) or dendritic spines (arrows), together with some extracellular clusters (asterisks). HA-tagged Ecrg4 also colocalized with green fluorescent protein (GFP)-tagged neuropeptide Y (NPY) (Figure 7—figure supplement 3B, C), suggesting transport in dense-core vesicles. Surface labeling of live cells also revealed clusters of Ecrg4 on axonal boutons and dendritic spines (Figure 7—figure supplement 3D, E), supporting active exocytosis and tethering via its binding partners to the plasma membrane. Exogenous application of the synthetic Ecrg4 peptide enhanced spine turnover (Figure 7—figure supplement 3F, G). Collectively, these observations suggest that the intracellular machinery for transporting and releasing Ecrg4 may be actively involved in regulating synapse formation and maturation.

### Rescue of the synaptic phenotype in schizophrenia-associated mouse models by *Ecrg4* suppression

**Figure 8.**
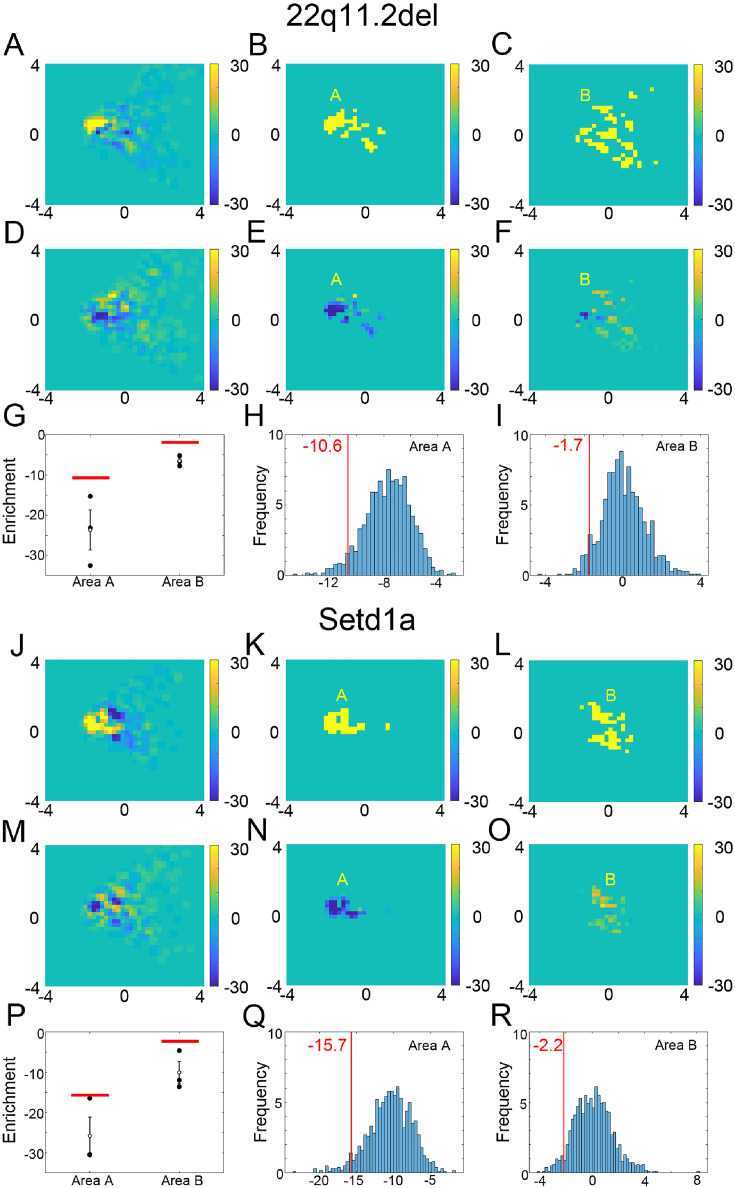
Altered spine population profiles after suppression of *Ecrg4* expression in neurons derived from two schizophrenia-associated mouse models: the 22q11.2^del/+^ mouse model (**A–I**) and *Setd1a*^+/−^ mouse model (**J–R**). (**A, J**) Density plot obtained by subtracting wild-type spines from mutant spines. Both wild-type and mutant neurons expressed control shRNA. (**B, K**) Area enriched with the mutant spines under the control shRNA condition. (**C, L**) Area depleted of the mutant spines under the control shRNA condition. (**D, M**) Density plot obtained by subtraction of the wild-type spines under control shRNA from the mutant spines under *Ecrg4* shRNA. This subtraction illustrates the normalizing effect of *Ecrg4* shRNA. (**E, N**) Reduction of mutant spines in area A by *Ecrg4* shRNA. The density plot, obtained by subtracting the mutant spines under control shRNA from those under *Ecrg4* shRNA, shows the extent of normalization by *Ecrg4* shRNA. (**F, O**) The increase of mutant spines in area B by *Ecrg4* shRNA using the same subtraction method. (**G, P**) The extent of rescue by *Ecrg4* shRNA in areas A and B for three independent culture experiments using either 22q11.2^del/+^ or *Setd1a*^+/−^ mouse models. The red lines show the values corresponding to statistical significance (p = 0.05), estimated from permutation analyses shown in (**H, I, Q, R**). (**H, Q**) Permutation analysis for area A to estimate the 95th percentile of the shuffled data. (**I, R**) Permutation analysis for area B.

The spatial localization of Ecrg4 protein in wild-type neurons and upregulated gene expression in the hippocampus of schizophrenia model mice strongly suggest a contribution to the observed spine abnormalities. To examine this possibility, we tested whether *Ecrg4* suppression by *Ecrg4*-shRNA rescues the synaptic phenotype of neurons derived from schizophrenia-associated mouse models (Figure 7—figure supplement 2H, I). We introduced a GFP expression plasmid along with the *Ecrg4*-shRNA plasmid or the control shRNA plasmid into neurons derived from 22q11.2^del/+^ and *Setd1a*^+/−^ mice (Supplementary file 1). The spine density plots generated from GFP-expressing neurons differed from those generated from DiI-labeled neurons. This difference may be due to the properties of SIM images obtained from either cell-surface DiI labeling or cytoplasmic GFP expression. Nevertheless, the subtracted (differential) distribution patterns between mutant and wild-type spines under the control shRNA condition indicate the enrichment of small spines (Figure 8A, J). From these plots, we identified the area showing enrichment of mutant spines (Figure 8B, K) and that of wild-type spines (Figure 8C, L). The pattern of small spine enrichment in mutant neurons shown in Figure 8A, J was no longer present in the plots generated by the subtraction of the wild-type spines under control shRNA from the mutant spines under *Ecrg4* shRNA, indicating the possible rescue of the phenotype (Figure 8D, M). Consistent with this interpretation, enrichment in both the mutant- and wild-type-dominant regions was attenuated (Figure 8E, F, N, O).

To determine whether the effect of *Ecrg4* shRNA exceeded that expected from random variation in spine counts, we performed a permutation analysis. Spine identities were randomly reassigned between the two conditions while preserving the total number of spines in each dataset, and the differential density maps were recalculated for each permutation. This procedure generated a null distribution of enrichment values expected under random assignment (Figure 8H, I, Q, R). The level of attenuation observed in both 22q11.2^del/+^ and *Setd1a*^+/−^ neurons exceeded the 95th percentile of the permuted distributions (p < 0.05), indicating that the obtained data were unlikely to be explained by stochastic variation alone (Figure 8G, P). This analysis supports the idea that *Ecrg4* downregulation can rescue the spine-shape impairment observed in schizophrenia-related mouse models.

## Discussion

We developed an objective method for identifying population-level differences in spine nanostructures and applied it to cultured hippocampal neurons derived from multiple mouse models of psychiatric disorders. We identified two distinct groups of mouse models based on population-level spine properties. Notably, these two objectively identified groups correspond to mouse models with ASD- and schizophrenia-related gene mutations. A higher fraction of the small spine population was a common characteristic of schizophrenia-associated mouse models. Time-lapse imaging of spine dynamics revealed that this smaller spine phenotype is associated with a reduced initial size and growth of spines and their enhanced turnover. Screening for genes differentially expressed between mutant and control mice identified *Ecrg4* overexpression as a potential molecular mechanism contributing to the schizophrenia spine phenotype. Indeed, suppression of *Ecrg4* expression normalized the spine phenotype in neurons derived from schizophrenia-associated mouse models. These findings support the use of population-level analysis of spine nanostructures as a powerful approach to elucidating the heterogeneous synaptic impairments underlying psychiatric disorders.

Spine nanostructures provide information about developmental history, prior activity, and current functional properties, but these fine structural details were not accessible by conventional light microscopy. However, recent advances in super-resolution microscopy allow for the study of nanoscale spine morphology, including in living preparations, and have revealed new structural features related to important synaptic functions (Tønnesen et al., 2014; Kashiwagi et al., 2019; Zaccard et al., 2020). Another challenge to spine nanoscale imaging is the structural heterogeneity across large spine populations, which may obscure important population-level differences. Nonetheless, to understand the physiology and pathology of neural networks, it is essential to obtain comprehensive information about a large number of spines within a single neuron and across local neuronal networks. We previously reported a dimensional reduction technique coupled with machine learning for evaluating spine properties at the population level (Kashiwagi et al., 2019), and have further refined this method for the current investigation to extract population-level nanostructural features of spines in multiple mouse models of psychiatric disorders. This method enabled us to compare spine phenotypes across different mouse models objectively and group disease models based on similarities and differences in the spine population. Notably, the two identified groups corresponded to ASD- and schizophrenia-related gene mutations, supporting the idea that population-level analyses of spine nanostructures provide meaningful information on synaptic abnormalities and dysfunction.

Population-level spine properties were more homogeneous in schizophrenia models (those with gene mutations implicated in schizophrenia) than in the other four models studied, in part due to a shared tendency for smaller spines. This observation is consistent with previous studies on *Setd1a* mutant mice, which showed reduced spine width, decreased mushroom-type spines, and lower spine density in the prefrontal cortex (Nagahama et al., 2020; Chen et al., 2022; Mukai et al., 2019). In contrast to these findings, several previous studies reported reduced numbers of small spines in the postmortem cortical tissues of schizophrenia patients (Obi-Nagata et al., 2023; MacDonald et al., 2017). However, this discrepancy may be attributed to the developmental period, as we examined fetal neuronal spines after 3 weeks of in vitro differentiation, whereas the aforementioned studies included postmortem samples from patients over 40 years of age. One possible scenario that may help unify these observations is that spine density is reduced in the early postnatal period, and that this reduction triggers homeostatic plasticity, which, in turn, reduces the fraction of small spines in later life. Another potential explanation is that schizophrenia differentially affects regional spine properties (e.g., hippocampus vs. cortex). Previous in vivo two-photon imaging studies reported higher spine turnover in the hippocampus than the cortex (Pfeiffer et al., 2018; Attardo et al., 2015). Therefore, the spine phenotype identified in this study may be specific to hippocampal pyramidal neurons. Finally, while the spine phenotype identified in the human postmortem brain undoubtedly resulted from complex interactions among genetic background, environmental influences, and regulation by non-neuronal cells, data from pure neuronal cultures are more likely to reflect the direct effects of schizophrenia-related gene mutations on synaptic functions. This property may be advantageous for identifying synaptic molecules that regulate synapse phenotypes in schizophrenia-related mouse models. However, the phenotype observed in the culture system requires confirmation using in vivo experiments of mouse models or human tissue samples. Efficient in vitro screening combined with reliable in vivo evaluation of synapses will facilitate translational research on mental disorders.

In addition to *Ecrg4*, these analyses identified several other DEGs common to mice harboring schizophrenia-related mutations. However, time-lapse imaging identified *Ecrg4* as the most effective spine regulator, and we also found that *Ecrg4* suppression reversed the abnormal spine phenotype in neurons derived from schizophrenia model mice. *Ecrg4* encodes a precursor protein of hormone-like peptides (Moriguchi et al., 2018). Exogenously expressed Ecrg4 exhibited a punctate pattern in both axonal and dendritic compartments, with a fraction of clusters present on the cell surface. This observation indicates that Ecrg4 may be secreted locally by axons or dendrites, thereby influencing synapses and adjacent neurons. Several signaling pathways act downstream of Ecrg4, including the stress-related NF-κB pathway and PI3K/Akt/mTOR pathway (Richter et al., 2023). Ecrg4-derived peptides may also act through Toll-like receptor 4 (TLR4) or multiple scavenger receptors, such as LOX1 (Moriguchi et al., 2018). While Ecrg4-related signaling pathways have been studied primarily in the context of inflammation and infection, there are few studies on Ecrg4-related signaling pathways in neurons. However, an *Ecrg4*-deficient mouse model demonstrated enhancements in neural stem cell proliferation and spatial learning, suggesting that overexpression may disrupt hippocampal function (Nakatani et al., 2019). We suggest that Ecrg4 may be a critical regulator of neural development and synaptogenesis in the hippocampus. Further studies are warranted to identify Ecrg4 receptors and downstream signaling pathways and to investigate the potential dysfunction of these pathways in schizophrenia-associated mouse models.

The nanoscale features of dendritic spines in mouse models of *Nlgn3*^R451C/(y or R451C)^, *Syngap1*^+/−^, *Pogz*^Q1038R/+^, and 15q11-13^dup/+^, which we classified as being related to ASD, are highly heterogeneous. This heterogeneity may reflect the broad clinical spectrum of ASD, which ranges from mild impairments in social skills to severe intellectual disability. Accordingly, these four mouse models may represent distinct subgroups characterized by different degrees or forms of hippocampal dysfunction. Notably, among the ASD-related models, 15q11-13^dup/+^ showed population-level spine properties closer to those found in the 22q11.2^del/+^ and *Setd1a*^+/−^ mouse models. Although we classified 22q11.2^del/+^ and *Setd1a*^+/−^ as schizophrenia-related models, both 22q11.2 deletion syndrome and *Setd1a* haploinsufficiency in humans are also associated with ASD, suggesting substantial overlap in the genetic risk factors underlying ASD and schizophrenia. Further systematic analyses linking rare genetic variants to synaptic phenotypes in mouse models may provide important insights into the mechanisms underlying both shared and disorder-specific synaptic alterations in neurodevelopmental and psychiatric disorders.

The most important advantage of our analytical method is reproducibility, despite the substantial heterogeneity in genotype and phenotype among these mouse models. Thus, we are confident that the spine population analysis described here will help researchers in different laboratories obtain reliable and comprehensive information on spine properties. Further, this method could facilitate comparisons across a large number of mouse models harboring mutations associated with mental disorders. Spine morphology as measured in a reduced culture system can also demonstrate direct effects of gene mutations on neuronal phenotypes in the absence of indirect influences from non-neuronal cells or specific environments. Ultimately, these studies may identify the shared and specific pathogenic mechanisms of psychiatric disorders and lead to improved diagnosis and treatments.

## Materials and methods

### Neuronal culture and genotyping of tissue samples from genetically modified mice

The following mouse models of psychiatric disorders were examined for spine nanostructural characteristics: male hemizygous or female homozygous neuroligin-3 R451C mutant (*Nlgn3*^R451C/(y or R451C)^), heterozygous *Syngap1* mutant mice (*Syngap1*^+/−^), heterozygous *Pogz* mutant mice (*Pogz*^Q1038R/+^), mice with duplication of chromosome 7C (15q11-13^dup/+^; corresponding to human 15q11-13 duplication), mice heterozygous for deletion on chromosome 16B2,3 (3q29^del/+^; corresponding to human 3q29 deletion), mice heterozygous for deletion on chromosome 16qA13 (22q11.2^del/+^; corresponding to 1.5 Mb deletion on human 22q11.2), mice heterozygous for *Setd1a* mutation (*Setd1a*^+/−^), and Ca^2+^/calmodulin-dependent protein kinase IIα kinase-dead mutant mice (*Camk2a*^K42R/K42R^). All animal experiments were reviewed and approved by the Institutional Animal Care and Use Committee of the University of Tokyo (permission number A2023M031-02).

For the production of embryos harboring heterozygous mutations, male heterozygous mutants were bred with wild-type female C57BL/6J or C57BL/6N mice (Japan SLC). Because neuronal cultures were prepared from wild-type and mutant littermates sharing the same genetic background, differences between the C57BL/6J and C57BL/6N sub-strains are unlikely to have affected the analysis. For the production of *Nlgn3*^R451C/(y or R451C)^ embryos, *Nlgn3*^R451C/y^ males and *Nlgn3*^R451C/+^ females were crossed. Embryos of *Camk2a*^K42R/K42R^ mice were obtained by crossing heterozygous *Camk2a*^K42R/+^ males and females. Both male and female embryos were used for the primary culture. Control cultures were prepared from littermate embryos without disease-related mutations on the background of either C57BL/6J or C57BL/6N.

Genomic DNA was purified from E16 embryos prior to brain dissection using the QuickGene DNA tissue kit (Wako), and genotypes were determined by PCR using either KapaTaq (Kapa Biosystems) or KOD FX Neo (TOYOBO) following the standard protocols provided by the manufacturers. The primer sequences used for genotyping were as follows:

*Nlgn3*^R451C/(y or R451C)^ genotyping:
Sense primer: 5′-GGTCAGAGCTGTCATTGTCAC-3′
Antisense primer: 5′-TGTACCAGGAATGGGAAGCAG-3′
*Syngap1*^+/−^ genotyping:
Sense primer for WT: 5′-GTCAGTGGGACATGGAAGTAG-3′
Sense primer for mutant: 5′-CTTCCTCGTGCTTTACGGTATC-3′
Antisense primer (common): 5′-CTGATCAGCCTGTCAGCAATG-3′
*Pogz*^Q1038R/+^ genotyping:
Sense primer for WT: 5′-TCTGTGAAGAAGCTTCGGGTAGTAC-3′
Antisense primer for WT: 5′-GTCTCCTCATTTACAGGGAGCT-3′
Sense primer for mutant: 5′-GCAGCGGCTCCCCGTTAAC-3′
Antisense primer for mutant: 5′-AGCGCACAGCCCACTCATAG-3′
15q11-13^dup/+^ genotyping:
Sense primer: 5′-AGAGGAGGGCCTTACTAATTACTTA-3′
Antisense primer: 5′-ATATGTACTTTTGCATATAGTATAC-3′
3q29^del/+^ genotyping:
Sense primer for WT: 5′-TTGGCACCACTCGCCCAAGTTATATCCACC-3′
Sense primer for mutant: 5′-CAAGGGGGAGGATTGGGAAGACAATAGCAG-3′
Antisense primer (common): 5′-GGTCATGCAAATTCTAGCAGTGAGTCATGAC-3′
22q11.2^del/+^ genotyping:
Sense primer for WT: 5′-GAGAAAGTGGGTGGGAAGGC-3′
Antisense primer for WT: 5′-GTCCCTCGCCACAGTCATAA-3′
Sense primer for mutant: 5′-CTAAGGAATGGTTCCGGCCA-3′
Antisense primer for mutant: 5′-TTTCACGGAGGCGGTATTCA-3′
*Setd1a*^+/−^ genotyping:
Sense primer: 5′-CTCGCCGCCATTTCTCTACATC-3′
Antisense primer: 5′-GTTCTGGAGGTTCTGGAGGTG-3′
*Camk2a*^K42R/K42R^ genotyping:
Sense primer: 5′-GGTCTTGAAGACTGTCTGGTGTGAGA-3′
Antisense primer: 5′-CACAGGCCAGTTTAGGTCTTGCTAGG-3′

After genotyping, primary hippocampal neuron cultures were prepared from mutant and corresponding control embryonic samples as described previously (Kashiwagi et al., 2019; Okabe et al., 1999). In brief, E16 hippocampi were dissected, minced, treated with trypsin (Gibco) and DNase (Sigma), and dissociated mechanically into a cell suspension. Cells were resuspended in minimum essential medium supplemented with B18 supplement, L-glutamine (Gibco), and 5% fetal calf serum (Equitech-Bio), and plated onto glass-bottom dishes (MatTek, #1.5) precoated with poly-L-lysine (Sigma). Glial proliferation was prevented by adding 5 μM Ara-C (Sigma) 2 days after plating. For the comparison of spine nanostructure between wild-type and mutant neurons, culture samples were processed for imaging at 18–22 DIV. For comparison of 8 mutant mouse models using SIM imaging, all imaging data were obtained from the samples fixed at 19 DIV. For pharmacological treatment, 10 μM CNQX or 20 μM bicuculline was applied to the culture medium at 13 DIV, and samples were processed for imaging at 19 DIV.

### Transfection and fluorescence labeling of primary hippocampal neurons

Primary neurons were transfected with the indicated vectors using the calcium phosphate precipitation method at 8–9 DIV according to a previously described procedure (Shin et al., 2013). All fluorescent proteins were expressed under the control of the β-actin promoter.

The expression of the shRNA constructs was induced using the pSilencer vector system. Briefly, the 19-nt target sequence and the reverse complement sequence of the target were ligated into the shRNA expression vector separated by a 9-nt spacer loop (TCTCTTGAA) (pSilencer 2.0-U6; Invitrogen). Stealth RNAi Negative Control Duplexes (Invitrogen) were used as control shRNAs. The targeting sequences for mouse *Met*, *Arhgap15*, and *Ecrg4* are as follows:

*Met* RNAi sequence:
5′-GCAGTGAATTAGTTCGCTA-3
*Arhgap15* RNAi sequence:
5′-AAGACAGATGTGAACATAC-3
*Ecrg4* RNAi sequence:
5′-GAGGCTAAATTTGAAGAT-3′

The suppression of target protein expression by these transfected shRNAs was tested by immunoblotting lysates of COS-7 cells co-transfected with a target gene construct fused to a sequence encoding amino acids 98–106 of human influenza hemagglutinin (HA), all under the control of the β-actin promoter. Overexpression of *Cip*4, *Ecrg4*, and *Npas4*, genes upregulated in cultures derived from mouse models of schizophrenia, was induced by transfection of plasmids containing the corresponding HA-fused cDNA under the control of the β-actin promoter. Briefly, mouse Trip10 transcript variant 4 (*Cip*4; NM_001242391) and mouse *Ecrg4* (NM_024283) were cloned, and an HA tag was inserted into the C-termini. Mouse *Npas4* (NM_153553) was cloned according to a previous report (Ooe et al., 2004) and fused with a Flag-HA tag at its C-terminus. Human NPY tagged with GFP (a kind gift from Dr. J. Takaska, NIH) was expressed under the control of the β-actin promoter.

For cell-surface labeling of individual neurons in culture, cells were fixed and stained with 1,1′-dioctadecyl-3,3,3′,3′-tetramethylindocarbocyanine perchlorate (DiI; Molecular Probes) as described previously (Kashiwagi et al., 2019).

### Immunocytochemistry

Cultured hippocampal neurons expressing HA-tagged constructs were fixed with 2% paraformaldehyde in PBS, permeabilized with 0.2% Triton X-100 in PBS, blocked with 5% normal goat serum, and reacted with rabbit anti-HA antibody (1:500; MBL), mouse anti-HA antibody (1:100; BioLegend), mouse anti-β-galactosidase antibody (1:500, Sigma), rabbit anti-Ecrg4 (1:100, Sigma), mouse anti-GM130 antibody (1:100, BD Biosciences), rabbit anti-Cip4/TRIP10 antibody (1:100, Proteintech), rabbit anti-NPAS4 antibody (1:100, Activity Signaling), and chicken anti-MAP2 antibody (1:500, PhosphoSolutions). After washing with PBS, samples were reacted with Alexa Fluor 546-labeled goat anti-rabbit IgG antibody (1:500; Invitrogen) and Alexa Fluor 647-labeled goat anti-mouse IgG antibody or anti-chicken IgY antibodies (1:500; Invitrogen). For cell-surface labeling with GFP, neurons expressing Ecrg4-HA-SEP (superecliptic pHluorin) were reacted with anti-GFP VHH/nanobody conjugated to Alexa Fluor 647 (1:4,000; Chromotek) in culture medium at 37°C.

### SIM imaging

For 3D-SIM imaging, we used an inverted microscope (ECLIPSE Ti-E, Nikon) equipped with an N-SIM-E system, 405, 488, 561, and 640 nm diode lasers (LU-NV, Nikon), and an oil-immersion TIRF objective lens (SR Apo TIRF 100×, N.A. 1.49, Nikon). All SIM images were acquired under strictly controlled conditions, including a stage temperature of 28–29°C, to minimize the position and aberration fluctuations. Spherical aberrations induced by refractive index mismatch were reduced by manually adjusting the objective lens correction collar. Images were acquired using an EMCCD camera (iXon3 DU-897E, Andor Technology) with 512 × 512 pixels (each pixel, 16 μm square) operated in the read-out mode at 1 MHz with 16-bit analog-to-digital conversion of the EM gain. All image-processing steps were performed in three dimensions. The acquired image datasets were computationally reconstructed using the reconstruction stack algorithm V2.10 of NIS-Elements AR (Nikon). An image stack consisted of 63 axial (*z*) planes of 512 × 512 pixels in the *x*- and *y*-dimensions, with a pixel size of 32 × 32 nm. All 3D images were reconstructed in 120 nm z-steps spanning 7.56 μm in the *z*-axis. The voxel size met the Nyquist criterion. Only dendritic segments spatially isolated from other fluorescent objects were selected for analysis because objects brighter than the targeted dendritic segments made image thresholding unreliable. Quality checks of the acquired SIM images were performed as described previously using the SIMcheck plugin for ImageJ (Ball et al., 2015).

### Spine isolation and shape measurement

Spines were isolated by automated image thresholding using a previously described method (Kashiwagi et al., 2019). Briefly, the reconstructed SIM image stacks were first processed by multilevel thresholding using the MATLAB built-in function [multithresh()] to produce binary images, which were further processed by geodesic active contours using the MATLAB built-in function [activecontour()]. The binary image stacks generated by thresholding the SIM images were then processed for the automated detection of spines. First, the dendritic shafts were fitted with elliptic cylinders and voxel clusters outside the best-fit elliptic cylinders were identified as spine candidates. Next, the spine candidates were classified by volume and shape. After selection, the junction between the dendritic shaft and spine was determined. Finally, the nanostructural parameters of isolated spines were measured. These steps are basically the same as those in our previous algorithm, but several points were improved, as described in the following sections. (The original code for SIM image processing is available at https://shigeookabe.github.io/download-page-SIM/ with a password of ‘simspineimage’.)

#### Dendritic shaft fitting

We first estimated the shaft volume using the custom-made function ‘SIM_Fitdendrite’ as previously described. In the new algorithm, the candidate spines were subsequently evaluated to determine whether their bases were large and spread on the shaft surface. If the spine base was large, the basal voxels were removed using the function ‘SIM_Removevoxel’.

#### Selection of the spines

Using the custom-made function ‘SIM_Position’, spines with volumes too small, too elongated, or located at the image edge were removed as previously described. Small spine candidates with volumes less than twice the volume of a rectangular cuboid with edge lengths equal to the theoretical resolutions of SIM images (<0.01 μm^3^) were rejected.

#### Adjustment of the spine–shaft junction

We determined the location of spine–shaft junction using the custom-made function ‘SIM_Junction’. This step was identical to our previous algorithm. Next, spines with more than one connection site to the shaft were detected using the function ‘SIM_Neck’ and eliminated from further analysis.

#### Measurement of nanostructural parameters

First, the angle of the spines was adjusted to be perpendicular to the long axis of the dendritic shaft and within the *xy*-plane using the function ‘SIM_Angle’. Next, nanostructural parameters were measured using the function ‘SIM_Shape’. In our previous spine-shape measurement study, a polygon mesh-based calculation was performed. In the current study, we took a different approach using voxel-based measurement. The calculation of structural parameters using voxel data was more straightforward and required fewer calculation steps than the method based on polygon mesh data. Spines were divided into 160 nm segments along the long axis using the function ‘SIM_Shape’. These spine segments were used to calculate the following 64 nanostructural parameters: spine length, surface area, base surface area, total volume, volume of each spine segment (20 segments), convex hull volume of each spine segment (20 segments), and convex hull ratio of each spine segment (20 segments).

### Analysis of the spine population data

Differences in spine phenotype among model mice were analyzed at the population level by PCA. We selected five parameters (spine length, base surface, total volume, and volumes of the 5th and 15th spine segments) for the PCA-based dimensional reduction. Two parameters that reflect the principal structural features (length and volume) were first selected. Second, three other parameters (base surface, volumes of the 5th and 15th spine segments) that were mutually independent and also independent from the first two parameters were chosen (pairwise correlation coefficients <0.3). The initial 64 parameters included the volume of each spine segment (20 segments), the convex hull volume of each spine segment (20 segments), and the convex hull ratio of each spine segment (20 segments). These parameters showed higher correlation coefficients but did not improve PCA performance. We also confirmed that PCA using all 64 parameters yields a cross-correlation map similar to that shown in Figure 2B. The first three principal components (PC1–PC3) covered approximately 90% of the variance in the data.

The 2D presentation of spine population data using PC1 and PC2 reflected the overall pattern of the spine-shape distribution, with thin, mushroom, and stubby spines separated in the feature space. We found that the overall pattern of spine population data projected to the PC1–PC2 plane differs between DiI-labeled neurons and GFP-expressing neurons. This difference may be due to the properties of SIM images obtained from either cell-surface labeling or cytoplasmic GFP expression. To facilitate the direct comparison of the spine population data in Figure 3 (DiI-labeled neurons) and Figure 8 (GFP-expressing neurons), we transformed the raw data of GFP-expressing neurons using the weight matrix calculated from the data for DiI-labeled neurons.

After PCA, relative spine densities (spine number per area in the feature space) were mapped onto an 80 × 80 grid spanning ±4 standard deviations along PC1 and PC2. Using this procedure, two density plots were generated: one from culture samples of disease model mice and the other from corresponding control cultures. The two density plots were then subtracted to extract the local differences in spine numbers. Because each grid contains spines with similar structural parameters, the subtracted plot reveals regions of mutant-dominant and wild-type-dominant spine distributions in the PC1–PC2 plane.

The similarity between the two subtracted density plots was evaluated by calculating the 2D cross-correlation *C* using the following equation:

$$\displaystyle  C=\frac{\sum \limits_{m=1}^{M}\sum \limits_{n=1}^{N}X\left (m,n\right)Y\left (m,n\right)}{\sqrt{\sum \limits_{m=1}^{M}\sum \limits_{n=1}^{N}X\left (m,n\right)X\left (m,n\right)\, }\sqrt{\sum \limits_{m=1}^{M}\sum \limits_{n=1}^{N}Y\left (m,n\right)Y\left (m,n\right)}},$$

where *X* and *Y* correspond to the two subtracted density matrices. The 11 different subtracted density plots shown in Figure 2 were clustered using the MATLAB function ‘linkage’, and the average distance between all pairs of elements in the two samples was calculated using the cluster algorithm.

For Figure 3, the PC1–PC2 feature space was divided into four subareas with distinct shape properties: (1) small and short, (2) small and long, (3) large and short, and (4) large and long. The median spine volume and length were first calculated from the wild-type population, and spine samples were classified into four subgroups based on these median values. Each position of the 80 × 80 grid was then labeled according to the dominant subgroup of spines it contained, yielding a map of four subareas corresponding to the four spine-shape categories.

In Figures 3 and 8, Figure 3—figure supplement 1, areas in the feature space enriched with mutant or control spines were extracted by searching the 80×80grid and identifying those where the difference in relative density was larger than the mean plus 3 × SD. Spine axial profiles in Figure 3—figure supplement 3 were then analyzed within areas enriched with either mutant or control spines.

### Time-lapse imaging of dendritic spines and image analysis

Live cell 3D imaging was performed using an A1 confocal laser scanning microscope (Nikon) equipped with 405, 488, 561, and 640 nm diode lasers (LU-NV, Nikon). Cells at 15–18 DIV were maintained at 37°C under a continuous flow of 5% CO_2_ to maintain medium pH using a heater stage system (INUG2H-TIZSH, Tokai Hit) with a lid designed for a glass-bottom dish to minimize evaporation. Horizontal and vertical drifts were controlled by a motorized stage and a perfect focus system, respectively. Images were acquired with an oil immersion objective lens (60×, NA 1.4, Nikon) at a confocal aperture of 1 AU. The imaged volume was 30.7 × 30.7 × 7.56 μm in *x*, *y*, and *z* directions, respectively, and image stacks consisted of 17 planes separated by 500 nm. Time-lapse images were recorded either at short intervals (15 min intervals for 24 h) or long intervals (two time points separated by 24 hr). Cultured neurons imaged at a 24-hr interval were then fixed and immunostained with anti-HA to confirm the expression levels of the transfected genes. The effect of exogenously applied Ecrg4 peptide (CΔ16; the C-terminal ECRG4 fragment of 16 aa, 5.4 μM, BEX Co, Ltd) was evaluated by taking time-lapse images at 24-hr intervals, with the first image taken just before the peptide application.

The image stacks obtained by time-lapse imaging were analyzed using custom-made MATLAB scripts. First, 2D images were generated by maximal intensity projection of z-stack images. These 2D images were processed to transform the outlines of dendritic shafts to be aligned with straight lines. Spines were subsequently identified as objects extending beyond the straight edges of the dendritic shafts. Identified spines were segmented, and total fluorescence intensities were measured as a proxy for spine volume. We classified the spines as stable (present throughout the imaging period), newly formed (appearing within 24-hr imaging period), and eliminated (lost within 24-hr imaging period). Spine turnover rate was calculated as the sum of newly formed and eliminated spines during a 24-hr interval, normalized to the total number of spines present at the beginning and end of the interval. The resulting value was expressed as a percentage. For newly formed and eliminated spines, we generated temporal profiles of growth and shrinkage using the total fluorescence intensity values.

### RNA sequencing and gene expression analysis

Total RNA was isolated from cultured hippocampal neurons at 19 DIV using the RNeasy Mini Kit (Qiagen) with the RNase-Free DNase Set (Qiagen) according to the manufacturer’s protocols. The quality of the extracted RNA was initially assessed using a NanoDrop spectrophotometer, and the RNA integrity number score was calculated for each sample using a Bioanalyzer (Agilent Technologies). The yields were quantified using a Qubit Fluorometer (Thermo Fisher Scientific). Sequencing libraries were prepared using the NEBNext Ultra Directional RNA Library Prep Kit for directional libraries (New England BioLabs) and the KAPA HTP Library Preparation Kit (KAPA Biosystems) according to the manufacturers' protocols. Paired-end RNA-seq libraries were sequenced using the Illumina HiSeq and MGI DNBSEQ-G400 platforms. Three independent culture preparations were used for RNA sequencing of each mouse model and control library. Following quality control, the reads were aligned with the mouse reference genome GRCm38/mm10 (GenBank assembly ID: GCA_000001635.2). Expression levels of genes were analyzed based on the transcripts per million, and differential expression analysis was conducted using the DESeq2 package in R (Love et al., 2014). DEGs were selected according to an adjusted p < 0.05 and a log_2_-fold change greater than 0.5.

### Simulation of spine development

Spine growth was simulated using a dendrite model consisting of 1000 segments, each 0.25 μm in length (250 μm in total). The spine distribution was updated every 20 min over 10 days, covering the initial phase of spine development from 8 to 17 DIV. The initial probability of new spine generation was set at 7.5 × 10^−3^ per segment per hour and decreased thereafter in proportion to the accrued spine density increase (by the final 24 hr, the probability of spine generation [mean ± SD] was 3.2 × 10^−3^ ± 0.23 × 10^−3^/segment/hr). This probability function yielded an average of 1.2 ± 0.22 new spines per 10 μm of dendrite every 24 hr, in good agreement with the spine dynamics measured by time-lapse imaging from 15 to 18 DIV (1.3 ± 0.63 new spines per 10μm of dendrite every 24 hr). The model also set the increase in spine volume (V1) from 6.7 × 10^−3^ to 3.3 × 10^−2^ in 20min. Spines in the early growth phase were treated as unstable, with loss probability P1 ranging from 9.0 × 10^−3^ to 0.1 every 20 min. Spines were modeled as stabilized when the individual volume reached a relative value of 1.0. These stabilized spines then entered a shrinking phase with a probability P2 ranging from 2.9 × 10^−3^ to 4.0 × 10^−3^ in 20 min. The shrinking rate V2 was set from 6.7 × 10^−3^ to 3.3 × 10^−2^ per 20 min. In simulations, the four parameters V1, V2, P1, and P2 were varied within these indicated ranges to find the combination producing the best fit with experimental data on the difference in spine lifetime distribution profile and turnover rate at 24 hr (the actual measurement interval). The simulation with the best-fit parameters yielded a spine density comparable to measured data (0.57 ± 0.029 vs. 0.70 ± 0.17 spines/μm of dendrite).

### Western blotting

Total protein extracts from cultured hippocampal neurons were separated into cytosolic and membrane fractions using Trident Membrane Protein Extraction Kit (GeneTex) according to the manufacturer’s protocol and separated on 10% sodium dodecyl sulfate (SDS)–polyacrylamide gels or 15–20% Tricine–SDS–polyacrylamide gels. The separated proteins were then transferred onto nitrocellulose membranes (Millipore) or PVDF membranes (Bio-Rad) using a wet transfer system. Membranes were blocked with 5% bovine serum albumin in Tris-buffered saline plus Tween-20 (TBS-T, 20 mM Tris-HCl, 137 mM NaCl, 0.2% Tween-20) for 1 hr at room temperature, then incubated overnight at 4°C with rabbit anti-Ecrg4 (1:1000, Sigma), rabbit anti-GluA1 (1:1000, Nittobo Medical), rabbit anti-GFP (1:3000, MBL), rabbit anti-dsRed2 (1:4000, Nittobo Medical), mouse anti-α-tubulin (1:3000, Sigma) as indicated. The membranes were washed and incubated with HRP-conjugated secondary antibody against mouse or rabbit IgG (1:10,000, Amersham) for 2 hr. Signals from the target protein bands were detected using a SuperSignal West Femto or Atto detection kit (Pierce) and imaged for quantitation (densitometry) using a ChemiDoc imaging system (Bio-Rad). We isolated the fraction of synaptosomes using Syn-PER Synaptic Protein Extraction Reagent (Thermo Scientific) in Figure 7—figure supplement 3A, according to the manufacturer’s protocols.

### Statistics

The statistical tests used for each experiment and the sample sizes (spines, dendrites, cells, and plates) are indicated in the corresponding text or figure legends. Statistical significance was determined as follows:

Figure 3G–J: One-way ANOVA followed by Tukey’s post hoc test.
Figure 5A–C: A linear mixed-effects model with genotype as a fixed effect and plate, cell, and dendrite as nested random effects.
Figure 5D–F: A linear mixed-effects model with genotype as a fixed effect and plate, cell, dendrite, and spine as nested random effects.
Figure 5G–L: A linear mixed-effects model with genotype as a fixed effect and plate, cell, dendrite, spine, and time as nested random effects.
Figure 7A: A linear mixed-effects model with treatment as a fixed effect and plate, cell, and dendrite as nested random effects.
Figure 8: Three independent imaging datasets evaluated by permutation tests.
Statistical significance was set at p < 0.05.

### Code availability

The original code for SIM image processing is available at https://shigeookabe.github.io/download-page-SIM/ with a password of ‘simspineimage’.

### Resource availability

Plasmid constructs for shRNA and HA-tagged protein expression are available upon request.

## Data Availability

The original code for SIM image processing is available at https://shigeookabe.github.io/download-page-SIM/ with a password of 'simspineimage'. The data is available from the following https://doi.org/10.5281/zenodo.21073564. Supplementary files 2 and 3 contain the data for differentially expressed genes (DEGs). The following dataset was generated: KashiwagiY QingruiL YasuhiroG RyoS AtsuA TakanobuN ShigeoO 2026Spine nanostructure profiling of cultured neurons from mouse models reveals a schizophrenia-linked role for Ecrg4Zenodo10.5281/zenodo.21073564PMC1346114842576600

## Additional files

Supplementary file 1. Structured illumination microscopy (SIM) imaging conditions. Information about mouse models, genetic background, culture data, genotype, number of cells analyzed, number of dendrites analyzed, the day of fixation after plating, and the presence or absence of manipulation. Data for eight disease models and for both 22q11.2^del/+^ and Setd1a^+/−^ with expression of shRNA for Ecrg4 were presented.

Supplementary file 2. Upregulated gene names Ensembl gene ID, gene symbols, log_2_-fold changes, and adjusted p values for upregulated genes found in culture preparations derived from eight disease models.

Supplementary file 3. Downregulated gene names Ensembl gene ID, gene symbols, log_2_-fold changes, and adjusted p values for downregulated genes found in culture preparations derived from eight disease models.

MDAR checklist

## Appendix 1

**Appendix 1—key resources table:**

Reagent type (species) or resource	Designation	Source or reference	Identifiers	Additional information
Strain, strain background (*Mus musculus*)	C57BL/6J	Japan SLC, Inc	N/A	
Strain, strain background (*M. musculus*)	C57BL/6N	Japan SLC, Inc	N/A	
Strain, strain background (*M. musculus*)	*Nlgn3* mutant	Tabuchi et al., 2007	PMID:17823315	Background: C57BL/6J Provided by Dr. Tabuchi
Strain, strain background (*M. musculus*)	*Syngap1* mutant	Komiyama et al., 2002	PMID:12427827	Background: C57BL/6J Provided by Dr. Grant
Strain, strain background (*M. musculus*)	*Pogz* mutant	Matsumura et al., 2020	PMID:32103003	Background: C57BL/6N Provided by Dr. Nakazawa
Strain, strain background (*M. musculus*)	15q11-13^dup/+^ mutant	Nakatani et al., 2009	PMID:19563756	Background: C57BL/6J Provided by Dr. Takumi
Strain, strain background (*M. musculus*)	3q29 mutant	Baba et al., 2019	PMID:31216562	Background: C57BL/6J Provided by Dr. Nakazawa
Strain, strain background (*M. musculus*)	22q11.2^del/+^ mutant	Saito et al., 2021	PMID:33845872	Background: C57BL/6N Provided by Dr. Aiba
Strain, strain background (*M. musculus*)	*Setd1a* mutant	Nagahama et al., 2020	PMID:32937141	Background: C57BL/6N Provided by Dr. Kano
Strain, strain background (*M. musculus*)	*Camk2a* mutant	Yamagata et al., 2009	PMID:19515929	Background: C57BL/6J Provided by Dr. Yamagata
Antibody	Anti-HA tag (rabbit polyclonal)	MBL International	Cat#561 RRID:AB_591839	1:500 in immunostaining
Antibody	Anti-HA tag (mouse, monoclonal)	BioLegend	Cat#901517 RRID:AB_2629622	1:100 in immunostaining
Antibody	Anti-β-galactosidase (mouse, monoclonal)	Sigma-Aldrich	Cat#G6282	1:500 in immunostaining
Antibody	Anti-Ecrg4 (rabbit, polyclonal)	BioLegend	Cat#901517 RRID:AB_2629622	1:100 in immunostaining; 1:1000 in immunoblot
Antibody	Anti-GM130 (mouse, monoclonal)	BD Biosciences	Cat#610823 RRID:AB_398142	1:100 in immunostaining
Antibody	Anti-TRIP10/Cip4 (rabbit, polyclonal)	Proteintech	Cat#10798-1-AP RRID:AB_2209248	1:100 in immunostaining
Antibody	Anti-NPAS4 (rabbit, monoclonal)	Activity Signalling	Cat#AS-AB18A RRID:AB_3678717	1:100 in immunostaining
Antibody	Anti-MAP2 (chicken, polyclonal)	PhosphoSolutions	Cat#1100-MAP2 RRID:AB_2492141	1:500 in immunostaining
Antibody	Alexa Fluor 546 conjugated anti-Rabbit IgG (goat, polyclonal)	Thermo Fisher Scientific	Cat#A-11035 RRID:AB_2534093	1:500 in immunostaining
Antibody	Alexa Fluor 647 conjugated anti-Rabbit IgG (goat, polyclonal)	Thermo Fisher Scientific	Cat#A-21235 RRID:AB_2535804	1:500 in immunostaining
Antibody	Alexa Fluor 647 conjugated anti-Chicken IgY (goat, polyclonal)	Thermo Fisher Scientific	Cat#A-21449 RRID:AB_2535866	1:500 in immunostaining
Antibody	Alexa Fluor 647 conjugated anti-GFP nanobody	Proteintech	Cat#gb2AF647 RRID:AB_2827575	1:4000 in immunostaining
Antibody	Anti-GFP (rabbit, polyclonal)	MBL International	Cat#598 RRID:AB_591819	1:3000 in immunoblot
Antibody	Anti-GluA1 (rabbit, polyclonal)	Nittobo Medical	Cat# MSFR102280 RRID:AB_2571752	1:1000 in immunoblot
Antibody	Anti-DsRed (rabbit, polyclonal)	Nittobo Medical	Cat#MSFR101400 RRID:AB_2571647	1:4000 in immunoblot
Antibody	Anti-α-tubulin (mouse, monoclonal)	Sigma-Aldrich	Cat#62204 RRID:AB_1965960	1:3000 in immunoblot
Antibody	ECL HRP-conjugated anti-mouse IgG (sheep, polyclonal)	Cytiva	Cat#NA931 RRID:AB_772210	1:10,000 in immunoblot
Antibody	ECL HRP-conjugated anti-rabbit IgG (donkey, polyclonal)	Cytiva	Cat#NA934 RRID:AB_772206	1:10,000 in immunoblot
Recombinant DNA reagent	*Cip*4-HA (plasmid)	This paper	N/A	Mouse *Trip10* (*Cip4*; NM_001242391)
Recombinant DNA reagent	*Npas4*-FH (plasmid)	This paper	N/A	Mouse *Npas4* (NM_153553)
Recombinant DNA reagent	*Ecrg4*-HA (plasmid)	This paper	N/A	Mouse *Ecrg4* (NM_024283)
Recombinant DNA reagent	*Ecrg4*-HA-SEP (plasmid)	This paper	N/A	SEP (superecliptic pHluorin) tagged mutant
Recombinant DNA reagent	*Met*-GFP (plasmid)	This paper	N/A	Mouse *Met* (NM_008591)
Recombinant DNA reagent	*Arhgap15*-GFP (plasmid)	This paper	N/A	Mouse *Arhgap15* (NM_153820)
Recombinant DNA reagent	NPY-GFP (plasmid)	Addgene	Cat#74629 RRID:Addgene_74629	Provided by Dr. Taraska
Recombinant DNA reagent	pSilencer-mouse-*Met* shRNA (plasmid)	This paper	N/A	5′-GCAGTGAATTAGTTCGCTA-3
Recombinant DNA reagent	pSilencer-mouse-*Arhgap15* shRNA (plasmid)	This paper	N/A	5′-AAGACAGATGTGAACATAC-3
Recombinant DNA reagent	pSilencer-mouse-*Ecrg4* shRNA (plasmid)	This paper	N/A	5′-GAGGCTAAATTTGAAGAT-3′
Peptide, recombinant protein	Ecrg4 peptide	BEX Co, Ltd	N/A	Ecrg4 C-terminal 16-mer peptide
Peptide, recombinant protein	Scramble peptide	BEX Co, Ltd	N/A	Corresponding scrambled control peptide
Commercial assay or kit	QuickGene DNA tissue kit	KURABO Industries Ltd	Cat#DT-S	
Commercial assay or kit	RNeasy Mini Kit	Qiagen	Cat#74104	
Commercial assay or kit	RNase-Free DNase Set	Qiagen	Cat#79254	
Commercial assay or kit	NEBNext Ultra Directional RNA Library Prep Kit	New England BioLabs	Cat#E7420	
Commercial assay or kit	KAPA HTP Library Preparation Kit	KAPA Biosystems	Cat#KK8234	
Commercial assay or kit	Trident Membrane Protein Extraction Kit	GeneTex International Corporation	Cat#GTX16373	For separation into cytosolic and membrane fractions
Commercial assay or kit	Syn-PER Synaptic Protein Extraction Reagent	Thermo Fisher Scientific	Cat#87793	For isolation of synaptosome fractions
Chemical compound, drug	CNQX	Tocris Bioscience	Cat#0190	
Chemical compound, drug	Bicuculline	Tocris Bioscience	Cat#0109	
Chemical compound, drug	DiI (1,1′-Dioctadecyl-3,3,3′,3′-Tetramethylindocarbocyanine Perchlorate)	Thermo Fisher Scientific	Cat#D3911	
Software, algorithm	SIM image processing (MATLAB)	This paper	N/A	https://shigeookabe.github.io/download-page-SIM/
Software, algorithm	DESeq2	Love et al., 2014	RRID:SCR_015687	
