# Supplementary material for: Spine nanostructure profiling of cultured neurons from mouse models reveals a schizophrenia-linked role for Ecrg4
Source: eLife. 2026 Aug 11;14:RP109083. doi: 10.7554/eLife.109083 (PMC13461148; doi:10.7554/eLife.109083)
Supplement: Supplementary file 3 [file elife-109083-fig7-figsupp3-data1.zip › Figure 7 - Figure supplement 3-source data 1/Figure 7—figure supplement 3 source data 1.pdf]

WB: Ecrg4

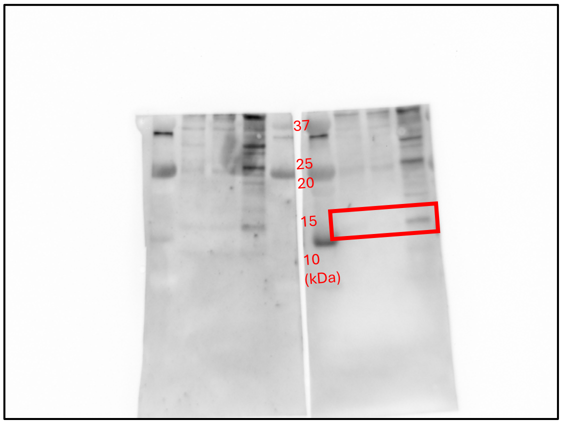

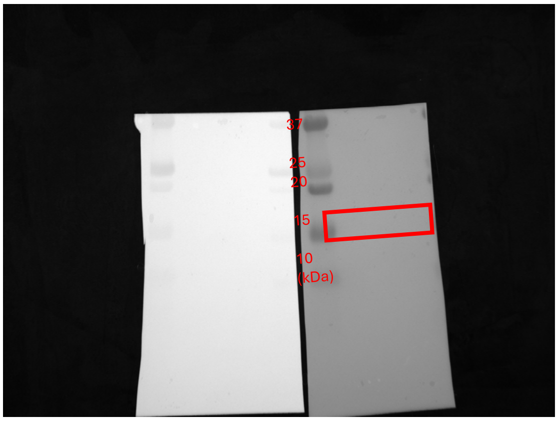

WB: GluA1

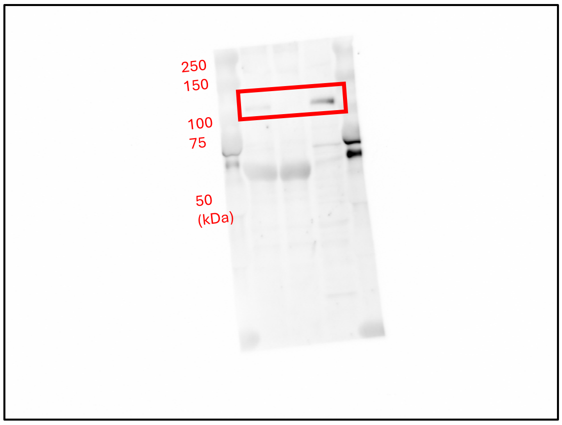

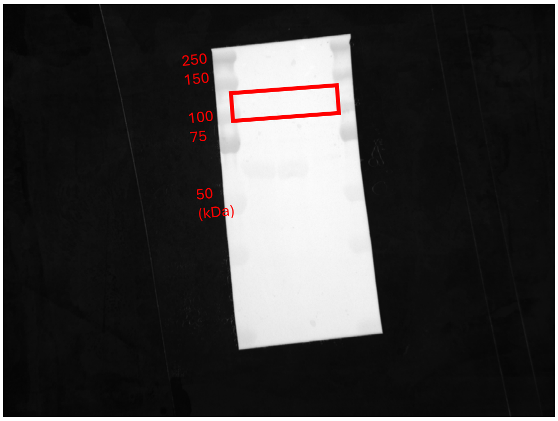

WB: α-tubulin

WB: GM130

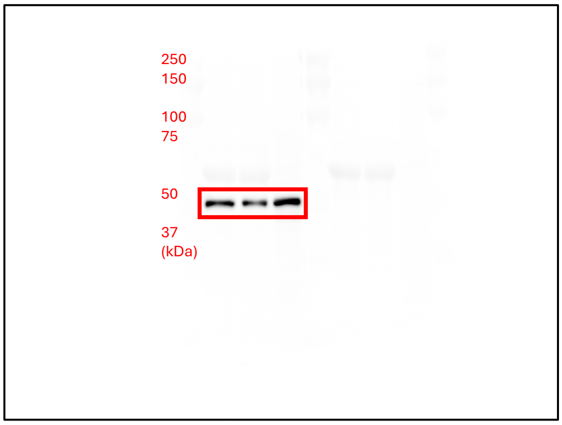

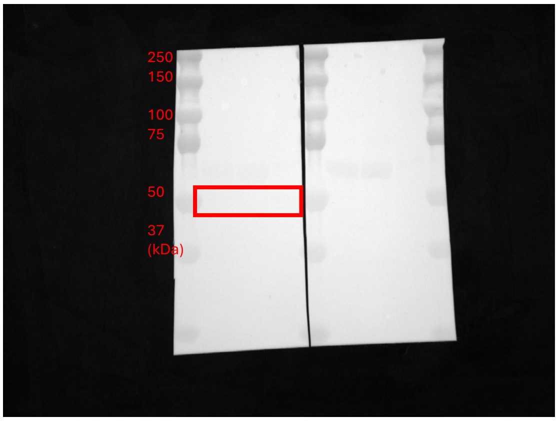

Figure 7- figure supplement 3, Source Data 1.
Original membranes corresponding to Figure 7- figure supplement 3 A. Precision Plus Protein^{TM} Dual Color Standards (BioRad) were employed.
