# Supplementary material for: Spine nanostructure profiling of cultured neurons from mouse models reveals a schizophrenia-linked role for Ecrg4
Source: eLife. 2026 Aug 11;14:RP109083. doi: 10.7554/eLife.109083 (PMC13461148; doi:10.7554/eLife.109083)
Supplement: Peer review history

# Editor report: eLife Assessment

**DOI:** 10.7554/eLife.109083.4.sa0

**Authors:** Jason P Lerch

**Affiliations:** University of Oxford United Kingdom

**Keywords:** Convincing, Important

By investigating spine nanostructure and dynamics across multiple genetic mouse models for neurodevelopmental disorders, this **important** study has the potential to uncover convergent or divergent synaptic phenotypes that may be specifically associated with autism versus schizophrenia risk. The imaging and overall breadth of the methods are **convincing**. The purely in vitro nature of the study slightly limits the generalisability of the findings, though these limitations are acknowledged and discussed in the manuscript.

# Referee report: Reviewer #1 (Public review)

**DOI:** 10.7554/eLife.109083.4.sa1

[Editors' note: this version has been assessed by the Reviewing Editor without further input from the original reviewers. The authors have addressed the comments raised in the previous round of review.]

Summary:

Kashiwagi et al. undertook a population analysis of dendritic spine nanostructure applied to the objective grouping of 8 mouse models of neuropsychiatric disorders. They report that spine morphology in cultured hippocampal neurons shows a higher similarity among schizophrenia mouse models (compared with autism spectrum disorder (ASD) mouse models) and identify an effect of Ecrg4 (encoding small secretory peptides) on spine dynamics and shape in these models.

Strengths:

The study developed a method for objectively comparing spine properties in primary hippocampal neuron cultures from 8 mouse models of psychiatric disorders at the population level using high-resolution structured illumination microscopy (SIM) imaging. This novel technique identified two distinct groups of mouse models according to the population-level spine properties: those with ASD-related gene mutations and those with schizophrenia-related gene mutations. Functional studies, including gene knockdown and overexpression experiments, identified an effect of Ecrg4 on the spine phenotype of the schizophrenia model mice.

Weaknesses:

The main weakness is that the study is wholly in vitro, using cultured hippocampal neurons. The authors present this as an advantage, however, arguing that spine morphology as measured in a reduced culture system can demonstrate direct effects of gene mutations on neuronal phenotypes in the absence of indirect influences from nonneuronal cells or specific environments.

# Referee report: Reviewer #2 (Public review)

**DOI:** 10.7554/eLife.109083.4.sa2

Okabe and colleagues build on a super-resolution-based technique they have previously developed in cultured hippocampal neurons, improving the pipeline and using it to analyze spine nanostructure differences across 8 different mouse lines with mutations in autism or schizophrenia (Sz) risk genes/pathways. It is a worthy goal to try to use multiple models to examine potential convergent (or not) phenotypes, and the authors have made a good selection of models. They identify some key differences between the autism versus the Sz risk gene models, primarily that dendritic spines are smaller in Sz models and (mostly) larger in autism risk gene models. They then focus on three models (2 Sz - 22q11.2 deletion, Setd1a; 1 ASD - Nlgn3) for timelapse imaging of spine dynamics, and together with computational modelling provide a mechanistic rationale for the smaller spines in Sz risk models. Bulk RNA sequencing of all 8 model cultures identifies several differentially expressed genes which they go on to test in cultures, finding that ecgr4 is upregulated in several Sz models and its misexpression recapitulates spine dynamics changes seen in the Sz mutants, while knockdown rescues spine dynamics changes in the Sz mutants. Overall, these have the potential to be very interesting findings and useful for the field.

# Author comment: Author response

**DOI:** 10.7554/eLife.109083.4.sa3

**Authors:** Shigeo Okabe, Yutaro Kashiwagi, Qingrui Liu, Yasuhiro Go, Ryo Saito, Atsu Aiba, Takanobu Nakazawa

**Affiliations:** The University of Tokyo Wako-shi Japan; The University of Tokyo Tokyo Japan; the University of Tokyo Tokyo Japan; University of Hyogo Kobe Japan; the University of Tokyo Tokyo Japan; The University of Tokyo Bunkyo-ku, Tokyo Japan; Tokyo University of Agriculture Tokyo Japan

The following is the authors’ response to the previous reviews

**Public Reviews:**

**Reviewer #2 (Public review):**

Okabe and colleagues build on a super-resolution-based technique they have previously developed in cultured hippocampal neurons, improving the pipeline and using it to analyze spine nanostructure differences across 8 different mouse lines with mutations in autism or schizophrenia (Sz) risk genes/pathways. It is a worthy goal to try to use multiple models to examine potential convergent (or not) phenotypes, and the authors have made a good selection of models. They identify some key differences between the autism versus the Sz risk gene models, primarily that dendritic spines are smaller in Sz models and (mostly) larger in autism risk gene models. They then focus on three models (2 Sz - 22q11.2 deletion, Setd1a; 1 ASD - Nlgn3) for time-lapse imaging of spine dynamics, and together with computational modelling provide a mechanistic rationale for the smaller spines in Sz risk models. Bulk RNA sequencing of all 8 model cultures identifies several differentially expressed genes which they go on to test in cultures, finding that ecgr4 is upregulated in several Sz models and its misexpression recapitulates spine dynamics changes seen in the Sz mutants, while knockdown rescues spine dynamics changes in the Sz mutants. Overall, these have the potential to be very interesting findings and useful for the field. My major concerns from the initial manuscript, especially regarding cherry picking and circularity have been addressed with revised analytical approaches. I have some remaining minor comments.

(1) The comparison between two wild-type samples versus wild-type-mutant samples is helpful - I think this could be added to the manuscript.

As suggested, we added the figure comparing two wild-type samples against wild-type mutant samples as Supplementary Figure 2.

(2) For results of time-lapse imaging - please spell out in the results section the direction of change (lines 270 - 277).

As suggested, we added the direction of change (an increase in the turnover rate) to the text (page 12, lines 270-271).

(3) Using linear mixed effect models for statistical analysis is a significant improvement. While a sample size (n) of mice = 3 is not ideal, I think given the multiple different mouse lines used and intensity of analysis, this is probably the best that can be done, although further validation in larger samples eventually is to be hoped for.

We appreciate the reviewer for recognizing the effort required to collect data across multiple mouse lines.

(4) The revised text is much improved, but I still think the authors should be upfront somewhere in the text that the schizophrenia-associated genes can only confer biased risk for schizophrenia (and that the clinical phenotype can also include autism). As I said before, I think this is the best we can do and I agree with their choices, but it is important not to overstate the link. The differences they see make it clear that these are still relevant distinctions.

As suggested by the reviewer, we further modified the discussion related to the comparison between ASD- and schizophrenia-associated mouse models (pages 23-24, lines 508-522).

“The nanoscale features of dendritic spines in mouse models of Nlgn3^R451C/(y or R451C)^, Syngap1^+/−^, POGZ^Q1038R/+^, and 15q11-13^dup/+^, which we classified as being related to ASD, are highly heterogeneous. This heterogeneity may reflect the broad clinical spectrum of ASD, which ranges from mild impairments in social skills to severe intellectual disability. Accordingly, these four mouse models may represent distinct subgroups characterized by different degrees or forms of hippocampal dysfunction. Notably, among the ASD-related models, 15q11-13^dup/+^ showed population-level spine properties closer to those found in the 22q11.2^del/+^ and Setd1a^+/-^ mouse models. Although we classified 22q11.2^del/+^ and Setd1a^+/-^ as schizophrenia-related models, both 22q11.2 deletion syndrome and Setd1a haploinsufficiency in humans are also associated with ASD, suggesting substantial overlap in the genetic risk factors underlying ASD and schizophrenia. Further systematic analyses linking rare genetic variants to synaptic phenotypes in mouse models may provide important insights into the mechanisms underlying both shared and disorder-specific synaptic alterations in neurodevelopmental and psychiatric disorders.”

**Recommendations for the authors:**

**Reviewer #2 (Recommendations for the authors):**

(1) I would suggest that it might be preferable to use the word 'neuropsychiatric' rather than 'mental' in the title.

As suggested, we modified the manuscript title.

(2) I think it would be clearer to say that DEGs are listed if present 'in three or more models' rather than >2 (I appreciate the latter is mathematically clear, but can easily be read as 2 or more if reading fast). This is changed in the figure legend, but I suggest it is also changed in the main text (line 352-3)

As suggested, we changed the main text to incorporate "in three or more models" (page 16, line 352).

(3) Please add to Methods (line 557) that 'control cultures were prepared from littermate embryos....'

As suggested, we added the phrase "control cultures were prepared from littermate embryos" (page 26, line 559).

(4) Sorry to add something, but please could the authors add a definition of how they calculate spine turnover (and add units to the y axis of Figure 5A-C)?

As suggested, we modified the y-axis of Figure 5A-C (% as unit) and added the method of calculating spine turnover rate in the text (page 36, lines 808-811).
